# A machine learning approach to personalized dose adjustment of lamotrigine using noninvasive clinical parameters

**DOI:** 10.1038/s41598-021-85157-x

**Published:** 2021-03-10

**Authors:** Xiuqing Zhu, Wencan Huang, Haoyang Lu, Zhanzhang Wang, Xiaojia Ni, Jinqing Hu, Shuhua Deng, Yaqian Tan, Lu Li, Ming Zhang, Chang Qiu, Yayan Luo, Hongzhen Chen, Shanqing Huang, Tao Xiao, Dewei Shang, Yuguan Wen

**Affiliations:** 1 Department of Pharmacy, The Affiliated Brain Hospital of Guangzhou Medical University (Guangzhou Huiai Hospital), Guangzhou, 510370 China; 2 Guangdong Engineering Technology Research Center for Translational Medicine of Mental Disorders, Guangzhou, 510370 China; 3 Department of Pharmacy, Guangzhou Bureau of Civil Affairs Psychiatric Hospital, Guangzhou, 510430 China; 4 Institute of Neuropsychiatry, The Affiliated Brain Hospital of Guangzhou Medical University (Guangzhou Huiai Hospital), Guangzhou, 510370 China

**Keywords:** Therapeutics, Adverse effects, Drug therapy, Epilepsy, Psychiatric disorders, Bipolar disorder, Depression, Psychosis, Schizophrenia, Data processing, Machine learning, Predictive medicine

## Abstract

The pharmacokinetic variability of lamotrigine (LTG) plays a significant role in its dosing requirements. Our goal here was to use noninvasive clinical parameters to predict the dose-adjusted concentrations (C/D ratio) of LTG based on machine learning (ML) algorithms. A total of 1141 therapeutic drug-monitoring measurements were used, 80% of which were randomly selected as the "derivation cohort" to develop the prediction algorithm, and the remaining 20% constituted the "validation cohort" to test the finally selected model. Fifteen ML models were optimized and evaluated by tenfold cross-validation on the "derivation cohort,” and were filtered by the mean absolute error (MAE). On the whole, the nonlinear models outperformed the linear models. The extra-trees’ regression algorithm delivered good performance, and was chosen to establish the predictive model. The important features were then analyzed and parameters of the model adjusted to develop the best prediction model, which accurately described the C/D ratio of LTG, especially in the intermediate-to-high range (≥ 22.1 μg mL^−1^ g^−1^ day), as illustrated by a minimal bias (mean relative error (%) =  + 3%), good precision (MAE = 8.7 μg mL^−1^ g^−1^ day), and a high percentage of predictions within ± 20% of the empirical values (60.47%). This is the first study, to the best of our knowledge, to use ML algorithms to predict the C/D ratio of LTG. The results here can help clinicians adjust doses of LTG administered to patients to minimize adverse reactions.

## Introduction

Lamotrigine (LTG) is a second-generation antiepileptic drug used commonly for monotherapy or adjunctive therapy for focal seizures, absence seizures, and generalized tonic–clonic seizures^1^. The drug has also been approved as a mood stabilizer for bipolar disorder to prevent mood relapses^2^. It is frequently associated with cutaneous adverse drug reactions ranging from mild maculopapular eruption to severe Stevens-Johnson syndrome and toxic epidermal necrolysis^3^. The incidence of toxicity increases in definite relation to rising concentrations of LTG^4^.

Pharmacokinetic variability, which can be influenced by age, pregnancy, drug-drug interactions, and concurrent diseases, plays a significant role in the dosing requirements for LTG^5^. Therapeutic drug monitoring (TDM), as an essential part of personalized medicine, is valuable for adjusting doses of LTG, and is especially recommended when other co-administered drugs that induce or inhibit the metabolism of LTG are prescribed or discontinued in the treatment regimen^6^. Thus, patients characterized by a combination of environmental and biological factors may benefit from TDM to adjust doses of LTG to minimize adverse reactions.

Dose-adjusted concentrations (C/D ratio), namely the ratio of drug concentration to dose under steady-state and trough conditions, is a parameter that can be easily obtained from TDM data. It can be calculated by dividing the trough steady-state concentration of a drug by the dose that the patient is taking^7^. A high C/D ratio indicates slow drug clearance while a low C/D ratio implies the reverse^7^. The C/D ratio has proven to be a valuable tool to facilitate dosing adjustment because it can be used to identify possible associations between adverse drug reactions and pharmacokinetic parameters^8^, analyze pharmacokinetic variability^9^, measure non-adherence to medication^10^, detect drug-drug interactions^11,12^, distinguish pharmacogenetic phenotypes (e.g., poor or ultra-rapid metabolizers)^12,13^, and estimate the dose required to achieve the desired concentration of the drug^13^.

Machine learning (ML) is defined as a field of study that enables computers to learn without being explicitly programmed^14^. It is a type of artificial intelligence that gives systems the ability to analyze a vast range of data collected from electronic health records (EHRs) and automatically learn from them using advanced statistical and probabilistic techniques to make more accurate predictions by constructing intelligent and effective predictive models^15^. Research in ML in the context of clinical medicine has revealed exciting advances in recent years, for example, the classification of medical images into diagnostic categories, detecting whether there are metastases on histological sections, and the automated segmentation of radiological images into known anatomical correlates to reduce diagnostics-related workload^16^. The application of ML to clinical drug therapies has garnered considerable research interest in recent years, and is playing an increasingly important role in the development of personalized dosing, especially in drug dose selection^17^. A few studies have been published on the application of ML to predict either drug doses or blood concentrations^18–23^. Jovanović et al.^18^ explored the application of ML as an alternative to pharmacokinetics analysis. A summary of the ML algorithms and drugs used, the purposes of prediction, sample sizes, and results are shown in Table 1.

**Table 1:** Summary of several relevant studies on models to predict either drug dosage or blood concentration.

Study	Algorithm	Drug	Purposes of prediction	Sample Size	Results
Jovanović^18^	CPANNs	Topiramate	Blood concentration	118 (78)^a^	RE (%) = 6.21, RMSE (%) = 39.9 for test set
Tang^19^	RT	Tacrolimus	Drug dosage	1 045	MAE = 0.73 mg/day and ideal rate (%) = 54.8 for validation cohorts^b^
Liu^20^	BART, MARS, and SVR	Warfarin	Drug dosage	4 798^d^	MAE (mg/week) for BART, MARS, and SVR were 8.87, 8.84, and 8.96, respectively, for validation cohorts Percentage within 20% for BART, MARS, and SVR were 46.03%, 46.35%, and 45.88%, respectively, for validation cohorts ^c^
Ma^21^	The stacked generalization ensemble frameworks (Stack 1 and 2)	Warfarin	Drug dosage	5 743 ^d^	MAE (mg/week) for Stack 1 and 2 were 8.31 and 8.31, respectively, for the hold-out test set. Percentage within 20% for Stack 1 and 2 were 47.85% and 47.81%, respectively, for the hold-out test set^c^
Roche-Lima^22^	RFR	Warfarin	Drug dosage	190	MAE = 4.73 mg/week and percentage within 20% = 80.56% for the overall test set
Chen^23^	CRT	Levothyroxine	Drug dosage	320	MAE = 13.0 μg and correct value (%) = 75 for evaluation set^e^

*CPANNs* counter-propagation artificial neural networks, *RT* regression tree, *BART* Bayesian additive regression trees, *MARS* multivariate adaptive regression splines, *SVR* support vector regression, *RFR* random forest regression, *CRT* classification and regression tree, *RE* relative error, *RMSE* root mean squared relative error, *MAE* mean absolute error.

^a^Therapeutic drug monitoring (TDM) measurements, with the number of patients in the parentheses.

^b^The ideal rate was defined as the percentage of patients for whom the predicted doses were within 20% of the actual stable therapeutic doses of tacrolimus.

^c^The percentage within 20% was defined as the percentage of patients for whom the predicted doses were within 20% of the actual stable therapeutic doses of warfarin.

^d^Data was obtained from the International Warfarin Pharmacogenetics Consortium (IWPC) open access dataset.

^e^The correct value (%) was defined as the percentage of patients for whom the predicted dose adjustment correctly fell within the smallest increment in doses of levothyroxine (12.5 mg).

The traditional pharmacokinetic analysis is based on mathematically simple techniques, for example, calculations of the area under the concentration–time curve (AUC). However, if the data are insufficient or cannot support a pharmacokinetic modeling approach, the model is inaccurate^24^. Recent years have seen growing interest in new statistical approaches, such as population pharmacokinetic (popPK) analysis, which can be used to extract useful information from sparse data^25^. A variety of programs are available for population modeling, of which nonlinear mixed-effects modeling (NONMEM) is the most widely used to analyze pharmacokinetic data^26^. However, building a popPK model usually requires understanding and choosing various mathematical models (e.g., a pharmacokinetic structure model related to dose, a population model for inter-subject variability, and a variance model for random residual variation), where this process is time consuming^27^. Furthermore, adding or removing a parameter may also be complicated owing to the explicit analytical model used^24^. In contrast, ML is known for its self-organizational and learning capabilities, which enables computers to learn from “experience” without being explicitly programmed^27^. Many previous studies have reported that ML algorithms, such as artificial neural networks (ANNs), have accuracies equivalent to or even higher than the NONMEM method^27,28^. Poynton et al.^29^ built an ensemble model by combining ANNs with NONMEM that was more accurate than either method.

ML algorithms fall into three categories: (1) supervised, (2) unsupervised, and (3) reinforcement learning. Supervised learning is used to predict classes or labels on unlabeled input data based on previously labeled data^30^. To date, the application of ML algorithms to predict the C/D ratio of LTG has not yet been reported. Therefore, in this study, we compare the performance of 15 supervised ML algorithms to identify the best one in terms of predicting the C/D ratio of LTG in Chinese patients based on noninvasive clinical parameters. We used clinical data to improve therapeutic efficacy while minimizing adverse effects. We also investigated clinical factors associated with the C/D ratio of LTG, and designed an easy-to-use web application as a real-time assisting clinical decision support tool for personalized adjustment to doses based on the proposed noninvasive predictive model. This is in the context of the costly implementation of the TDM because of the dedicated staff and equipment required^31^, which makes it unaffordable as a routine procedure in most hospitals in developing countries like China.

## Results

### Basic characteristics of the entire dataset

**Figure 1:**
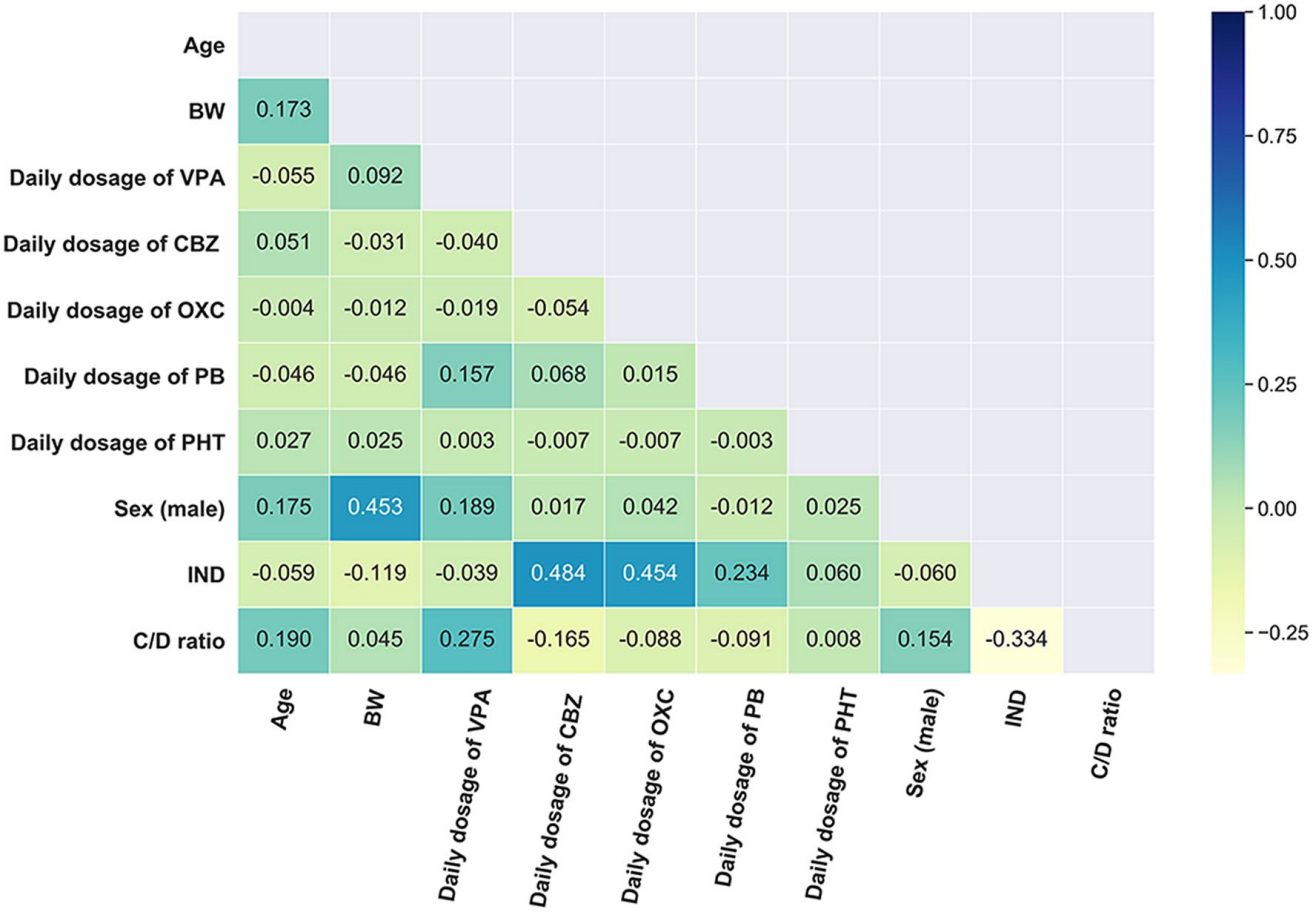
Heatmap visualization of the correlations between the dose-adjusted concentrations (C/D ratio) and the variables analyzed by Pearson’s correlation coefficient. *BW* body weight, *VPA* valproic acid, *CBZ* carbamazepine, *OXC* oxcarbazepine, *PB* phenobarbitone, *PHT* phenytoin, *IND* enzyme inducers.

In the dataset used, the mean values of the serum concentration and daily dose of LTG were 5.52 (range: 0.50–18.55) µg/mL and 171 (range: 12.5–500) mg/day, respectively. The mean value of the calculated C/D ratio parameter was 35 (range: 4.0–147) μg·mL^−1^·g^−1^·day. The histogram and quantile–quantile (Q-Q) plot of the C/D ratio are shown in Supplementary Fig. S1a,c, indicating that it was normally distributed. The distribution of features in the entire dataset is shown in Table 2. None of the variables had a missing rate (i.e., the percentage of missing values) above 50% in our dataset. Note that the daily dosage of a drug was assumed to be zero if it had not been taken. A heatmap of Pearson's correlations between the C/D ratio and the variables was shown in Fig. 1.

**Table 2:** Distribution of features in the entire dataset (N = 1141).

	Value	Distribution in the dataset (N, %)	Missing (N, %)
**Categorical variable**			
Gender	Male	659, 57.76%	0, 0%
	Female	482, 42.24%	0, 0%
Co-administration of IND	Yes	226, 19.81%	0, 0%
	No	915, 80.19%	0, 0%
**Continuous variable**			
Age (year)	34.88 (17.58)^a^	1 141, 100%	0, 0%
BW (kg)	60.33 (13.95)^a^	1 117, 97.90%	24, 2.10%
VPA daily dosage (mg)	250 (0–6912)^a^	981, 85.98%	160, 14.02%
CBZ daily dosage (mg)	0 (0–1200)^a^	1 141, 100%	0, 0%
OXC daily dosage (mg)	0 (0–1800)^a^	1 141, 100%	0, 0%
PB daily dosage (mg)	0 (0–300)^a^	1 141, 100%	0, 0%
PHT daily dosage (mg)	0 (0–100)^a^	1 141, 100%	0, 0%

*IND* enzyme inducers, *BW* body weight, *VPA* valproic acid, *CBZ* carbamazepine, *OXC* oxcarbazepine, *PB* phenobarbitone, *PHT* phenytoin.

^a^Continuous data are reported as mean values (standard deviation) or median values (minimum ~ maximum).

### Imputation evaluation

A total of 184 missing data points were imputed. An overall comparison of models using the entire dataset and the omitted dataset is shown in Table 3. When all features and default parameters of the models were used, the nonlinear models outperformed the linear models on the whole. The imputation generated a bias in both linear and nonlinear models. However, when the results of t-tests of the training set were omitted, the values of the mean absolute error (MAE) in the test set showed no statistical significance between the entire dataset and the omitted dataset in most nonlinear models, indicating that the imputation had a smaller impact on the nonlinear models. Thus, the entire dataset was considered in the modeling study.

**Table 3:** An overall comparison of mean absolute error (MAE) at 95% confidence intervals (CI) for the prediction of dose-adjusted concentrations of lamotrigine in the derivation cohort for models using two datasets.

Regression models	Entire dataset		Omitted dataset		*P* values (vs. entire dataset)*
	MAE [+ 95% CI, − 95% CI] μg mL^−1^ g^−1^ day		MAE [+ 95% CI, − 95% CI] μg mL^−1^ g^−1^·day		
	Training Set	Test Set	Training Set	Test Set	
**Linear models**					
MLR	13.4 [13.6, 13.3]	13.6[14.6, 12.6]	12.5 [12.7, 12.3]	12.7 [14.3, 11.1]	< 0.001; 0.009
RidgeR	13.5 [13.6, 13.3]	13.6 [14.6, 12.7]	12.6 [12.8, 12.3]	12.7 [14.5, 11.0]	< 0.001; 0.014
LSVR	13.9 [14.0, 13.7]	13.9 [15.4, 12.5]	12.9 [13.1, 12.7]	13.1 [15.5, 10.6]	< 0.001; 0.065
LassoR	15.2 [15.3, 15.0]	15.2 [17.0, 13.5]	14.0 [14.3, 13.7]	14.0 [16.8, 11.3]	< 0.001; 0.047
**Nonlinear models**					
ETR	2.3 [2.4, 2.1]	9.7 [11.5, 7.8]	2.4 [2.5, 2.3]	8.9 [10.2, 7.6]	0.004; 0.092
BR	4.9 [5.1, 4.8]	9.8 [11.8, 7.8]	4.7 [4.8, 4.5]	8.9[10.4, 7.4]	< 0.001; 0.054
RFR	4.9 [5.0, 4.8]	9.9[12.0, 7.9]	4.7 [4.9, 4.5]	9.1 [10.4, 7.7]	0.002; 0.052
GBR	8.3 [8.5, 8.1]	10.0[11.3, 8.7]	7.6 [7.8, 7.4]	9.3 [11.3, 7.4]	< 0.001; 0.120
XGBR	8.6 [8.7, 8.4]	10.0 [11.4, 8.6]	7.9 [8.1, 7.7]	9.5 [11.3, 7.7]	< 0.001; 0.227
KNR	8.9 [9.1, 8.7]	11.2[13.5, 8.8]	8.2 [8.4, 8.0]	10.0[12.0, 8.0]	< 0.001; 0.048
DTR	2.3 [2.4, 2.1]	10.9 [13.4, 8.4]	2.4 [2.5, 2.3]	10.0 [11.1, 9.0]	0.004; 0.061
MLPR	10.3 [10.9, 9.7]	11.1 [12.7, 9.5]	9.8 [10.1, 9.5]	10.8 [12.6, 8.9]	0.001; 0.489
ABR	13.2 [14.0, 12.3]	13.6 [14.8, 12.4]	11.8 [12.9, 10.8]	12.5 [14.3, 10.8]	< 0.001; 0.006
SVR (kernel = 'rbf')	14.5 [14.7, 14.4]	14.6 [16.3, 12.9]	13.4 [13.7, 13.1]	13.5 [16.4, 10.5]	< 0.001; 0.052
NuSVR (kernel = 'rbf')	14.8 [15.0, 14.7]	14.9 [16.6, 13.1]	13.6 [14.0, 13.3]	13.7 [16.5, 10.9]	< 0.001; 0.042

*MLR* multiple linear regression, *RidgeR* ridge regression, *LassoR* lasso regression, *LSVR* linear-support vector regression, *ETR* extra-trees’ regression, *KNR* k-nearest neighbor regression, *BR* bagging regression, *RFR* random forest regression, *XGBR* XGBoost regression, *GBR* gradient boosted regression, *DTR* decision tree regression, *SVR* support vector regression, *NuSVR* nu-support vector regression, *MLPR* multi-layer perceptron regression, *ABR* AdaBoost regression.

*P values for the training set (the left side of the semicolon) and the test set (the right side of the semicolon), respectively.

### Comparison of performance of models

An overall comparison of the optimized predictive regression models in the derivation cohort of the entire dataset is shown in Table 4. The grid search-based parameter optimization for each ML algorithm is listed in Supplementary Tables S1–S15. Overall, the nonlinear models were superior to the linear models. All nonlinear models yielded lower MAE values than the multiple linear regression (MLR) model (statistically significant). The t-tests for the MAE in the test set showed that linear models delivered the same performance at a 95% confidence interval (CI). Among the best nonlinear models, the extra-trees’ regression (ETR), k-nearest neighbor regression (KNR), bagging regression (BR), random forest regression (RFR), XGBoost regression (XGBR), gradient-boosted regression (GBR), and decision tree regression (DTR) models yielded similar values of the MAE in the test set, where the differences among them were not statistically significant (range: 9.4–10.1 μg·mL^−1^·g^−1^·day). The ETR algorithm, a tree-based ensemble algorithm, was chosen to establish the predictive model.

**Table 4:** The mean absolute error (MAE) at confidence intervals (CI) of 95% for the prediction of dose-adjusted concentrations of lamotrigine in the derivation cohort in the entire dataset for optimized regression models.

Regression models	MAE [+ 95% CI, − 95% CI] μg mL^−1^ g^−1^ day		*P* values (vs. MLR)*	*P* values (vs. ETR)*
	Training set	Test set		
**Linear models**				
MLR	13.5 [13.6, 13.3]	13.6 [14.7, 12.5]	-	< 0.001; < 0.001
RidgeR	13.6 [13.7, 13.4]	13.7 [14.7, 12.6]	0.005; 0.695	< 0.001; < 0.001
LassoR	13.5 [13.7, 13.3]	13.6 [14.7, 12.5]	0.210; 1.000	< 0.001; < 0.001
LSVR	13.6 [14.1, 13.2]	13.8 [14.7, 12.8]	0.023; 0.576	< 0.001; < 0.001
**Nonlinear models**				
ETR	5.4 [5.6, 5.3]	9.4 [11.2, 7.7]	< 0.001; < 0.001	-
KNR	2.4 [2.5, 2.2]	9.6 [11.6, 7.5]	< 0.001; < 0.001	< 0.001; 0.743
BR	5.8 [6.0, 5.6]	9.6 [11.7, 7.5]	< 0.001; < 0.001	< 0.001; 0.681
RFR	6.6 [6.8, 6.4]	9.6 [11.6, 7.7]	< 0.001; < 0.001	< 0.001; 0.634
XGBR	8.4 [8.6, 8.1]	9.9 [11.2, 8.5]	< 0.001; < 0.001	< 0.001; 0.234
GBR	7.9 [8.1, 7.8]	9.9 [11.4, 8.4]	< 0.001; < 0.001	< 0.001; 0.251
DTR	7.3 [7.7, 6.8]	10.1 [12.1, 8.1]	< 0.001; < 0.001	< 0.001; 0.142
SVR (kernel = 'rbf')	7.8 [8.0, 7.6]	10.3 [12.0, 8.7]	< 0.001; < 0.001	< 0.001; 0.034
NuSVR (kernel = 'rbf')	8.1 [8.4, 7.9]	10.3 [12.0, 8.6]	< 0.001; < 0.001	< 0.001; 0.038
MLPR	10.6 [10.9, 10.2]	11.0 [12.5, 9.5]	< 0.001; < 0.001	< 0.001; < 0.001
ABR	12.3 [12.4, 12.1]	12.5 [14.0, 10.9]	< 0.001; 0.002	< 0.001; < 0.001

*MLR* multiple linear regression, *RidgeR* ridge regression, *LassoR* lasso regression, *LSVR* linear-support vector regression, *ETR* extra-trees’ regression, *KNR* k-nearest neighbor regression, *BR* bagging regression, *RFR* random forest regression, *XGBR* XGBoost regression, *GBR* gradient boosted regression, *DTR* decision tree regression, *SVR* support vector regression, *NuSVR* nu-support vector regression, *MLPR* multi-layer perceptron regression, *ABR* AdaBoost regression.

*P values for the training set (the left side of the semicolon) and the test set (the right side of the semicolon), respectively.

### Optimizing ETR model

**Figure 2:**
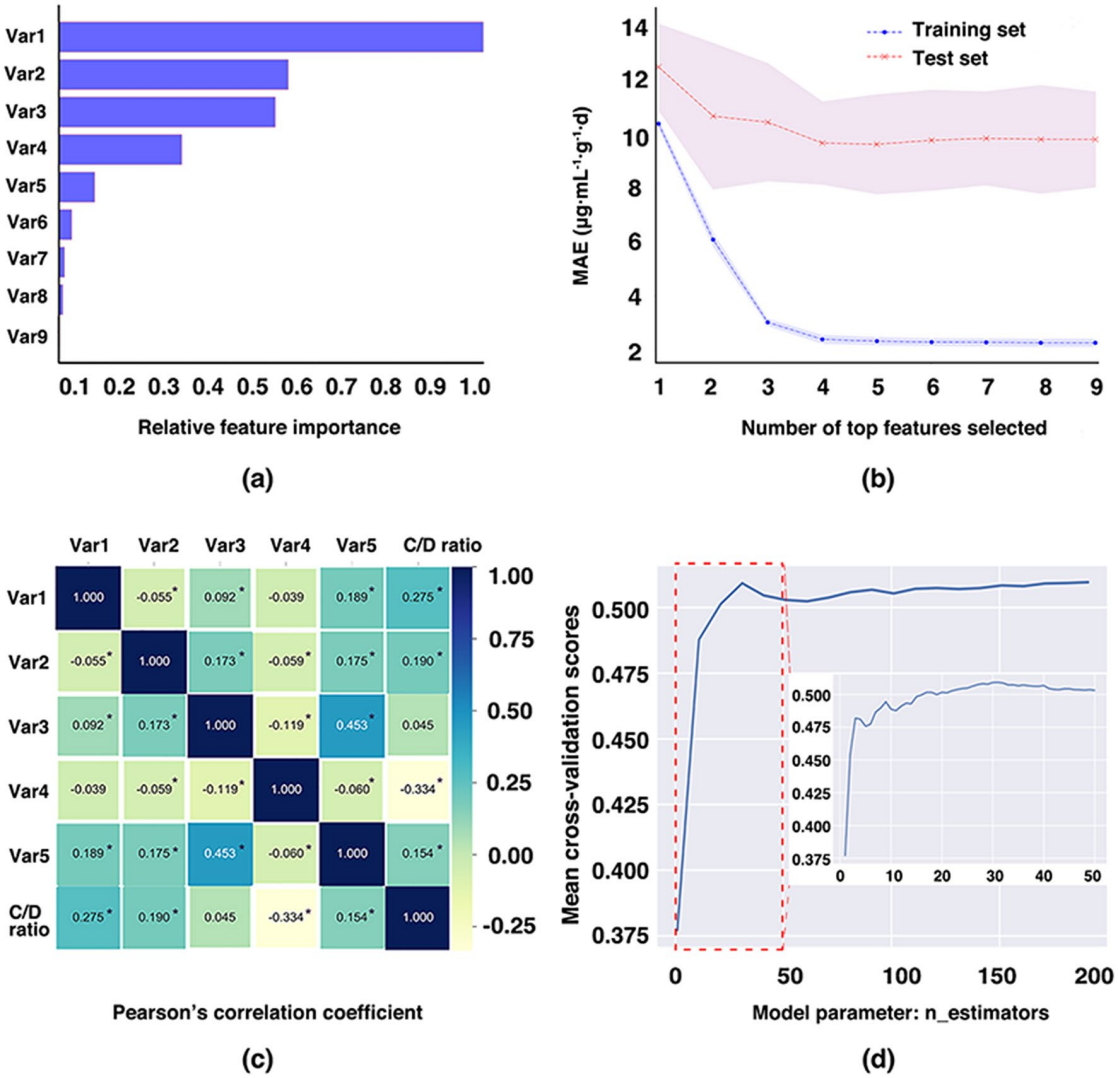
Optimization of the extra-trees’ regression model. (**a**) The features are ranked according to the relative importance for the prediction of dose-adjusted concentrations (C/D ratio) of lamotrigine based on the tree-based feature selection strategy. (**b**) The composition of the feature set (i.e., the top five features) is selected by the forward feature selection strategy. The colored area indicates the corresponding standard deviations obtained via tenfold cross-validation. (**c**) Heatmap visualization of the correlations between the C/D ratio and the selected features analyzed by Pearson’s correlation coefficient. “*” represents *P* values < 0.05. (**d**) The optimal value of the model parameter n_estimators is filtered by mean cross-validation scores in the test set using the forward-parameter selection strategy, by iteratively generating increasingly larger parameter values and the corresponding scores. The abbreviations used in the legends represent the following: Var1 [daily dosage of valproic acid (VPA)]; Var2 (age); Var3 [body weight (BW)]; Var4 [enzyme inducers (IND)]; Var5 [sex (male)]; Var6 [daily dosage of oxcarbazepine (OXC)]; Var7 [daily dosage of carbamazepine (CBZ)]; Var8 [daily dosage of phenobarbitone (PB)]; Var9 [daily dosage of phenytoin (PHT)].

The ETR algorithm was employed to select important features. A tree-based method and forward feature selection were applied to compute feature importance and discard irrelevant features. Figure 2a shows the results of the feature selection method. The daily dosage of valproic acid (VPA) appeared to be the most important determinant of the C/D ratio of LTG. The relative importance of all features for predicting the C/D ratio of LTG was ranked as follows: daily dosage of VPA (1.0000), age (0.5400), body weight (BW) (0.5096), enzyme inducers (IND) (0.2893), sex (male) (0.0842), daily dosage of oxcarbazepine (OXC) (0.0299), daily dosage of carbamazepine (CBZ) (0.0128), daily dosage of phenobarbitone (PB) (0.0089), and daily dosage of phenytoin (PHT) (0.0014).

To determine the optimal feature set, we identified the point at which there was no considerable change in the decline in MAE in the test set when a feature was added to the model. The results of the forward feature selection method showed that the MAE decreased gradually at first, as expected, and then tended to minimum values when the following features were used: daily dosage of VPA, age, BW, IND, and sex (male) (see Fig. 2b).

Further, Pearson’s correlation and a heat map analysis revealed weakly positive correlations between the C/D ratio, and the daily dosage of VPA (*r* = 0.275, *P* < 0.001), age (*r* = 0.190, *P* < 0.001), BW (*r* = 0.045, *P* = 0.130), and sex (male) (*r* = 0.154, *P* < 0.001); conversely, a moderately negative correlation was observed between the C/D ratio and IND (*r* = − 0.334, *P* < 0.001). The selected features were not found to be multi-collinear (see Fig. 2c).

We also adjusted the key parameter n_estimators, which can be considered to be the number of trees, as it had the most significant influence on the performance of the ETR algorithm but did not affect the complexity of any model. Based on the method used to choose the forward parameter using tenfold cross-validation, the maximum mean cross-validation score (0.5093) was achieved on the test set when the parameter n_estimators (default value = 10) was increased to 31 (see Fig. 2d). Adjustments to other key parameters yielded higher scores when n_estimators was set to 31 by using tenfold cross-validation-based grid search (see Supplementary Table S1). Below are the main parameters that were optimized for our final ETR model: (1) 'n_estimators': 31; (2) 'max_depth': 20; (3) 'min_samples_leaf': 1; (4) 'min_samples_split': 6; and (5) 'max_features': 'auto'.

We then compared the predictive performance of the ETR model before and after feature selection and parameter adjustment on the derivation cohort. The optimal ETR model enhanced generalization by reducing the overfitting yielded a lower MAE on the test set (not statistically significant, *P* = 0.106), but obtained a higher MAE on the training set (statistically significant, *P* < 0.001) than the ETR model before optimization.

### Validation and assessment of ETR model

**Figure 3:**
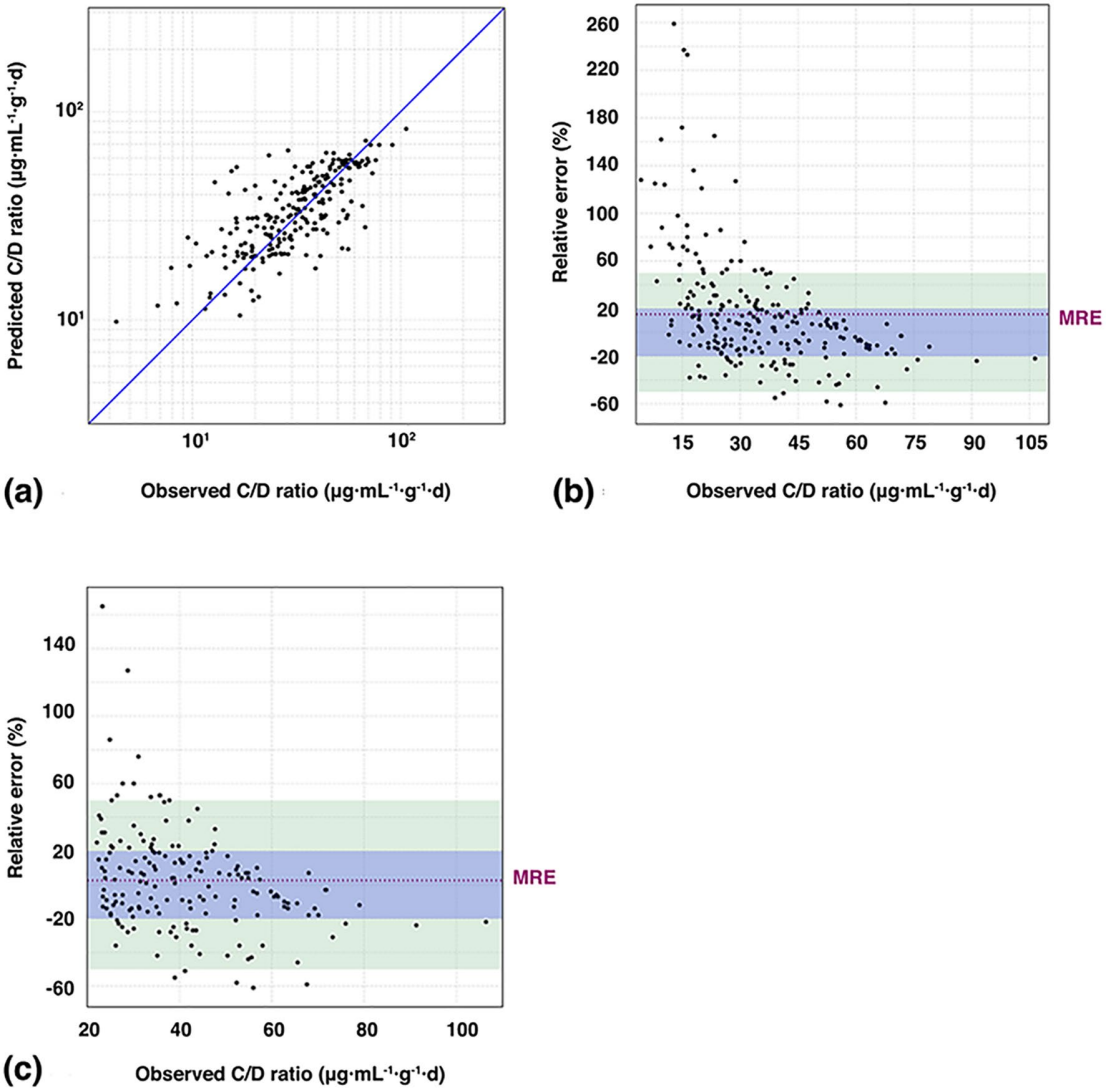
Scatter plots of the predicted vs. observed dose-adjusted concentrations (C/D ratio) and relative error (%) vs. observed C/D ratio. (**a**) Goodness-of-fit plot for model prediction of the C/D ratio. (**b**) Relative error (%) vs. observed C/D ratio over the entire range of the C/D ratio. (**c**) Relative error (%) vs. observed C/D ratio over the range ≥ 22.1 μg mL^−1^ g^−1^ day. The blue and green areas indicate ± 20% and ± 50% ranges, respectively. *MRE* mean relative error.

Finally, to determine the overall predictive performance of the chosen prediction model, three indices, namely the MAE, mean relative error (MRE) (%), and percentage within 20%, were applied to the validating cohort. As is shown in Table 5, the predictive model was able to accurately describe the C/D ratio of LTG, especially in the intermediate-to-high range (≥ 22.1 μg mL^−1^ g^−1^ day), as illustrated by a minimal bias (MRE (%) =  + 3%) and good precision (MAE = 8.7 μg mL^−1^ g^−1^ day). About 60.47% of all relative errors versus the observed C/D ratio ≥ 22.1 μg mL^−1^ g^−1^ day were in the ± 20% range. Overall, the model provided more accurate predictions in the intermediate and high ranges of the C/D ratio than its low range (see Fig. 3).

**Table 5:** The total number of therapeutic drug monitoring (TDM) measurements, mean relative error (MRE) (%), mean absolute error (MAE), and percentages of predictions within 20% of the observed dose-adjusted concentrations (C/D ratio) in the validation cohort depending for different ranges of the C/D ratio in the context of modeling it and log_10_(C/D ratio) when treated as labels, respectively.

Items	Modeling labels	C/D ratio range		
		Total range	Low range (< 22.1 μg mL^−1^ g^−1^ day)	Intermediate-to-high range (≥ 22.1 μg mL^−1^ g^−1^ day)
Total number of TDM measurements		229	57	172
MRE (%)	C/D ratio	+ 15	+ 53	+ 3
	log_10_(C/D ratio)	+ 8	+ 43	− 4
MAE (μg mL^−1^ g^−1^ day)	C/D ratio	8.7	8.8	8.7
	log_10_(C/D ratio)	8.9	7.2	9.4
Percentage within 20% (%)	C/D ratio	53.71	33.33	60.47
	log_10_(C/D ratio)	51.53	39.66	55.56

We compared the effect of the predictive performance of the ETR models after optimization on the derivation cohort in the context of modeling the C/D ratio and its log_10_-transformed parameter [log_10_(C/D ratio)] when treated as labels. The log_10_(C/D ratio) [mean value (range): 1.48 (0.60–2.168) log_10_-μg mL^−1^ g^−1^ day] showed a normal distribution (see Supplementary Fig. S1b,d). The grid search-based parameter optimization for the ETR model in the context of modeling log_10_(C/D ratio) is listed in Supplementary Table S16. Overall, the performance of the ETR model in terms of predicting the C/D ratio was superior to that in terms of predicting log_10_(C/D ratio) in the intermediate and high ranges of the C/D ratio (see Table 5).

## Discussion

In addition to the popPK method^32^, other data-driven techniques have been used to predict the dose and/or concentration of LTG. For instance, Nakamura et al*.*^33^ reported significant linear relationships between the LTG concentrations in week 2 and those at week 8 for Japanese patients of depression, some of whom were being administered VPA while the others were not. They estimated an optimal dose of LTG by building regression equations. Yamamoto et al*.*^34^ studied factors influencing LTG concentrations in Japanese patients of epilepsy by stepwise multiple regression analysis that enabled them to estimate the LTG concentration by applying the coefficients of these factors to a multiple regression model. However, to the best of our knowledge, scarcely any study has used ML methods to predict the C/D ratio, where this is a key parameter for analyzing pharmacokinetic abnormalities^7^. Our study, therefore, fills this gap in the literature. Our present work here has improved on these abovementioned studies, as reflected in the following: First, even though datasets related to the LTG have been explored in these studies, the TDM measurements were not adequate. Moreover, these datasets did not include the trough concentrations used to calculate the C/D ratio or the co-administered drugs that induce the hepatic drug-metabolizing enzymes. However, our dataset was based on Chinese population, and had a larger number of samples of trough LTG concentrations and more comprehensive variables (including age, gender, BW, and IND). Second, unlike past work that has focused on linear regression models, our modeling study considered both linear and nonlinear regression models. ML can help detect the nonlinear and complex interactions between variables by minimizing the error between the predicted and the measured values^35^. Third, our models were trained and tested on the derivation cohort, and validated on another validation cohort randomly split from the entire dataset. Such external validation verifies the generalizability of our models to new data^36^. Finally, the superiority of ML in terms of developing predictive models becomes clearer on larger populations with greater numbers of predictors. The relevant factors may not be recognized owing to missing values. However, the ML method involves imputing these missing values^35^.

No one ML algorithm is the most accurate in all cases, and thus comparisons of ML algorithms in different fields of research, and on different datasets, may yield different results^37^. In this study, a comparison of the regression models showed that other linear models performed similarly on the test set to the MLR model, a frequently used and simple statistical model that makes it easy to interpret the variables^38^. A possible explanation for this is that these linear models had the same optimal feature set (see Supplementary Tables S12–S15), and no multi-collinear relationship was obtained between the features (see Fig. 1). Our results also indicate that the nonlinear models were statistically significantly stronger than the MLR model, which can be in part attributed to the weak linear correlations between consecutive parameters (see Fig. 1) and the high signal-to-noise ratio^35^. Compared with linear models, the nonlinear models did not require linear relationships between the parameters. Moreover, many factors affect the outputs, particularly in nonlinear, dynamic disease states. Therefore, the dataset may inevitably contain noise that can affect linear regression models more than nonlinear regression models^38^.

Better features mean flexible, simple models that yield good predictive results. A total of five patient variables were identified by the variable selection process of the extra-trees’ algorithm to develop the noninvasive predictive model, daily dosage of VPA, age, BW, IND, and sex, where the daily dosage of VPA and IND were identified as the most important positive and negative predictors, respectively. Uridine glucuronosyltransferases (UGTs) play an essential role in the metabolism of LTG. LTG dosage in adjunctive therapy is usually dependent on the interactions of LTG with co-administered drugs. An enzyme inhibitor is a type of drug that binds to the enzyme and reduces its metabolic activity. As a broad-spectrum enzyme inhibitor, the VPA can inhibit the metabolic activities of many hepatic drug-metabolizing enzymes to different extents^39^. An IND is a type of drug that binds to the enzyme, activates it, or increases its gene coding expression, and then increases its metabolic activity. Previous studies have reported that IND induces the metabolism of LTG, increases its clearance, and lowers its serum levels by 34–52%, whereas VPA inhibits the metabolism of LTG, decreases its apparent clearance by 38.5%, and raises its serum levels twofold^40–42^. A previous study revealed that the clearance of LTG increases to a maximum at 36 years and then gradually decreases in adult patients^43^. In general, in agreement with the earlier study, we found that the data points with a high C/D ratio were more likely to be distributed among younger or older patients (see Supplementary Fig. S2a), and the kernel density analysis showed that the data points with a low C/D ratio were concentrated in patients 20–30 years of age (see Supplementary Fig. S2b). Due to the difference in the gene coding expression or metabolic activity of the UGTs, sex may influence the pharmacokinetics of LTG^44^. VPA is known to have endocrinal side effects, and is likely to affect fertility^45^. Thus, young women likely tend to use other drugs instead. In the context of the co-administration of the VPA, the TDM measurements for men were 2.3 times those for women in our study (see Supplementary Fig. S2c). This can in part explain the weakly positive correlations between the C/D ratio of LTG, and age and sex (male) in our study. Brzaković et al.^44^ reported that BW may influence the interactions of LTG, and overweight patients may be less susceptible to these interactions. BW was identified as another important predictor for our model but showed an insignificant correlation with the C/D ratio via Pearson’s correlation analysis. This might be due to their nonlinear relationship (see Supplementary Fig. S2d), as Pearson's correlation coefficient is sensitive to only linear relationships.

**Figure 4:**
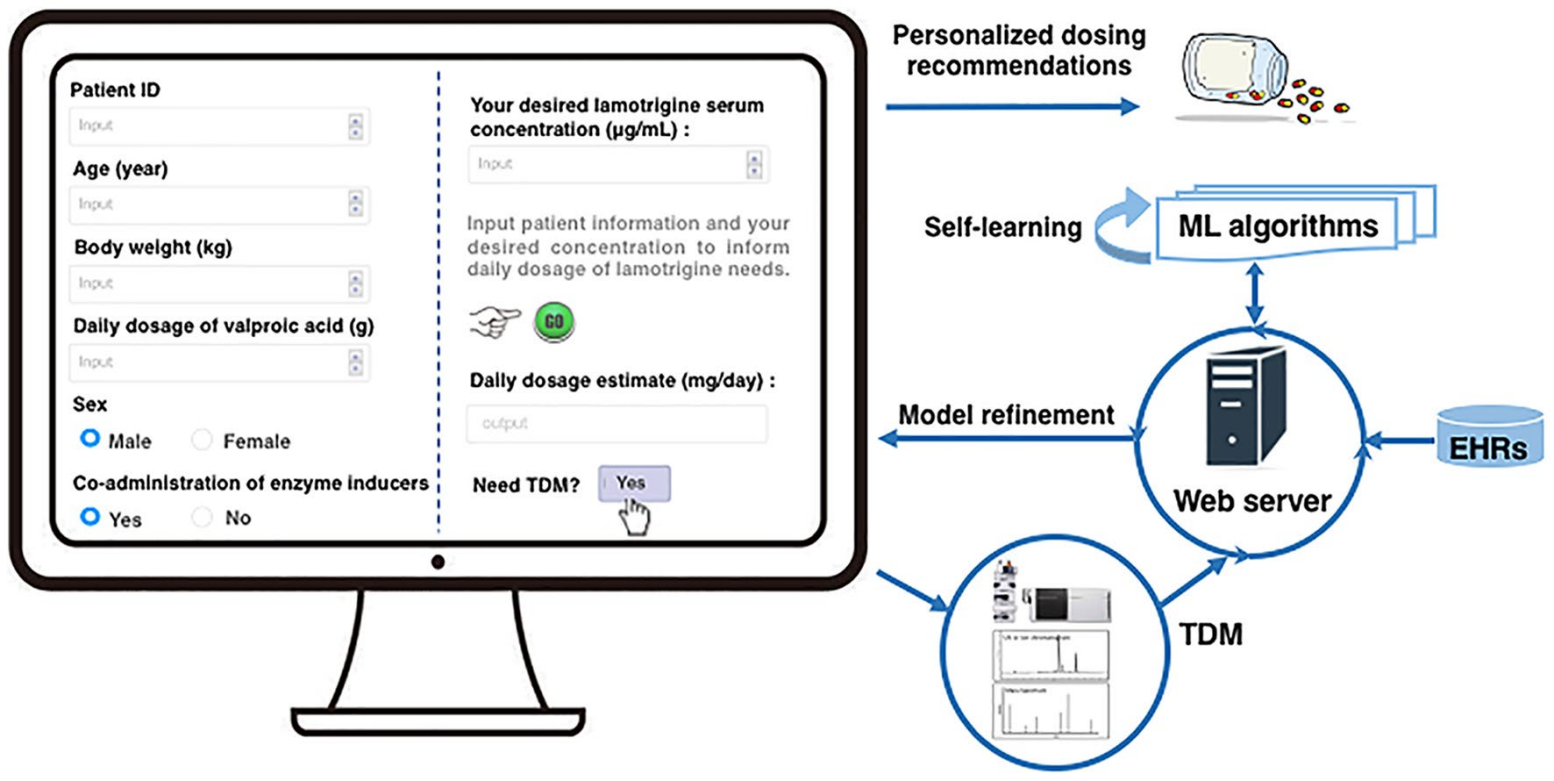
Web application to personalize dosing adjustments of lamotrigine, and the model's self-learning and refinement processes. *ML* machine learning, *TDM* therapeutic drug-monitoring, *EHRs* electronic health records.

The prediction model also showed a much higher variance at low C/D ratios, possibly due to the overfitting problem in the training set, as well as a greater influence of uncontrolled factors like genetic effects and TDM measurement errors. Even so, our prediction model in general performed better in the intermediate and high ranges of the C/D ratio (lower MAE and MRE (%), and a higher percentage within 20%). Moreover, patients in these ranges were more likely to benefit more from our model because a high C/D ratio indicated slow drug clearance^7^; hence, such patients may suffer from overdose and toxicity more easily. The analysis revealed that multiple factors (age, gender, BW, and co-administered drugs) had an impact on the C/D ratios of LTG. However, not all these factors were considered in the recommended dosing regimens according to the latest package inserts of LTG approved by the National Medical Products Administration (NMPA). For instance, the recommended usual maintenance dose for LTG monotherapy in patients of epilepsy older than 12 years is 100–200 mg/day, while a strict rate of dose escalation, starting at low doses, is recommended owing to the possibility of life-threatening rashes. To promote the clinical applications of our model, we designed an easy-to-use web application for personalized dose adjustments by allowing prescribers to input the characteristics of patients to estimate their LTG dosing needs. For example, according to the latest Arbeitsgemeinschaft für Neuropsychopharmakologie und Pharmakopsychiatrie (AGNP) guideline for TDM in neuropsychopharmacology^7^, the recommended therapeutic reference ranges for LTG are 3–15 μg/mL as an anticonvulsant drug and 1–6 μg/mL as a mood-stabilizing drug. Given that the C/D ratio was predicted to be 60 μg mL^−1^ g^−1^ day, an estimated maintenance dose of 50 mg/day (= 3/60 × 1 000 mg/day) was required for adult patients of epilepsy to reach 3 μg/mL, and 250 mg/day (= 15/60 × 1 000 mg/day) to reach 15 μg/mL (assuming good therapeutic responses)^7^. If the predicted C/D ratio was assumed to be 80 μg mL^−1^ g^−1^ day due to changes in the patients’ BW, ignoring problems of adherence and drug-drug interactions, estimated maintenance doses of 37.5 mg/day (= 3/80 × 1000 mg/day) and 187.5 mg/day (= 15/80 × 1 000 mg/day) were required to reach the target concentrations of 3 μg/mL and 15 μg/mL, respectively. Thus, unlike the dose recommended for most patients in the package inserts, this application can tailor doses for specific patients. Further, our model can be implemented within electronic health systems. Thus, once the TDM has been implemented, it automatically crawls the information on the characteristics of patients using their EHRs as well as the TDM measurements. Such implementation would make it easier to increase the size and variety of the dataset, in which case the ML algorithm has enough data to learn to generate robust predictions. More importantly, these designs, particularly the ML-based algorithm, can be applied to other drugs with the use of TDM highly recommended as a clinical routine for dose optimization. A snapshot of this application, and the model’s self-learning and optimizing process are depicted in Fig. 4.

Despite the promising results, there is room to improve the predictive models overall. Several key limitations of this study should be noted. First, the sample size used in our model was small, and a much larger number of samples is needed for further development and evaluation. In future work, using the abovementioned web application, we should be able to collect and store data more efficiently, and use big data to optimize the prediction model. Second, owing to the use of retrospective data instead of prospective in our study, certain uncontrollable factors were unavoidable. For example, the C/D ratios might have decreased due to nonadherence^46^. The inaccurate timing of the blood collection might also have led to the fluctuation in LTG concentration, especially in the case of the co-administration of IND^47^. The influence of inevitable experimental errors on the C/D ratios, especially its low range, could not be accurately evaluated either. Finally, the variables used in our model, though readily available, are all non-genetic factors. The effects of genetic polymorphisms on LTG pharmacokinetics have been widely explored in previous studies, but have yielded some conflicting findings^48,49^. Thus, more variables (e.g., genetic factors) needed to be included in the model. Despite these limitations, by using only noninvasive clinical parameters, the proposed model has the potential to help adjust doses of LTG and minimize adverse reactions. However, note that it is not an all-powerful tool in clinical practice because complex interactions (e.g., gene-environment) play a major role in many disorders, such as neurological and psychiatric disorders^50^. Hence, even though our model has achieved promising results, the best way of personalizing LTG doses is to monitor the serum concentration of LTG and attend to its clinical response.

## Conclusion

The results here proved the feasibility of ML algorithms, especially the nonlinear model, for predicting the C/D ratio of the LTG and identifying important predictors. By using the extra-trees’ regression algorithm (one of the best nonlinear models) and noninvasive clinical parameters (including the daily dosage of the VPA, age, BW, IND, and sex), the proposed predictive model delivered good performance, especially in the intermediate-to-high range of the C/D ratio of LTG (≥ 22.1 μg mL^−1^ g^−1^ day). Furthermore, an easy-to-use web application based on the proposed predictive model was designed as a real-time assisting clinical decision support tool to help with the personalized dose adjustment of LTG, thus improving its therapeutic efficacy while minimizing its adverse effects. Finally, the application of the proposed predictive model requires further improvement overall.

## Methods

### Dataset

This retrospective study was performed on 1141 TDM measurements obtained from 347 Chinese patients who had received LTG treatment at the Affiliated Brain Hospital of Guangzhou Medical University in 2018 and 2019. The patients were on monotherapy with LTG or concomitant therapy with VPA or IND (including CBZ, OXC, PB, and PHT). All patients’ data were collected from the EHRs during TDM, and included demographic characteristics (e.g., gender, age, and BW), LTG dosing regimens, TDM measurements, and co-medication status when the LTG concentration was determined. The data collection was approved by the independent Ethics Committee of the Affiliated Brain Hospital of Guangzhou Medical University ([2016] NO.060). The requirement for informed consent was waived by the Ethics Committee of the Affiliated Brain Hospital of Guangzhou Medical University that approved the study, owing to the retrospective nature of the analyses. This study was carried out in compliance with the guidelines of the Helsinki Declaration.

TDM measurements of LTG were considered for enrollment. If the patient was in a steady state (usually therapy with a stable dose for at least four to six half-lives^7^, trough sampling was performed before the next dosage, and the concentrations were not below the lower limit of quantification (LLQ). Each of the 1141 C/D ratios calculated, along with its corresponding covariates (including demographic and clinical information), was treated as a new input (i.e., feature) and output (i.e., label) data pair. The categorical covariates included sex (male or female) and the co-administration of IND (yes or no). The continuous covariates included age, BW, and daily dosages of co-administered drugs (including VPA, CBZ, OXC, PB, and PHT) when the concentration of LTG was determined.

### Bioanalysis

The serum concentration of LTG was determined by a validated, analytical, high-performance liquid chromatography (HPLC) method that included an HPLC system (Agilent 1260; Agilent Technologies, Inc., Santa Clara, USA). The serum samples were extracted by methanol as protein precipitators. Pirfenidone was taken as the internal standard. Eclipse Plus C_18_ column (150 mm × 4.6 mm, 5 μm) was used with the mobile phase of methanol: water (5 mmol/L ammonium formate) = 52: 48 (v/v) at a flow rate of 0.8 mL/min, a column temperature of 40 °C, detection wavelength of 310 nm, and injected volume of 10 μL. The standard curve was linear (*r*^2^ = 0.9933), and ranged from 0.5 μg/mL to 20 μg/mL. The LLQ was validated at 0.5 μg/mL. The mean extraction recoveries of the LTG from plasma at three quality control (QC) levels were found to be over 91.24%, whereas the intra- and inter-day precisions were both less than 11.54%.

### ML strategies

**Figure 5:**
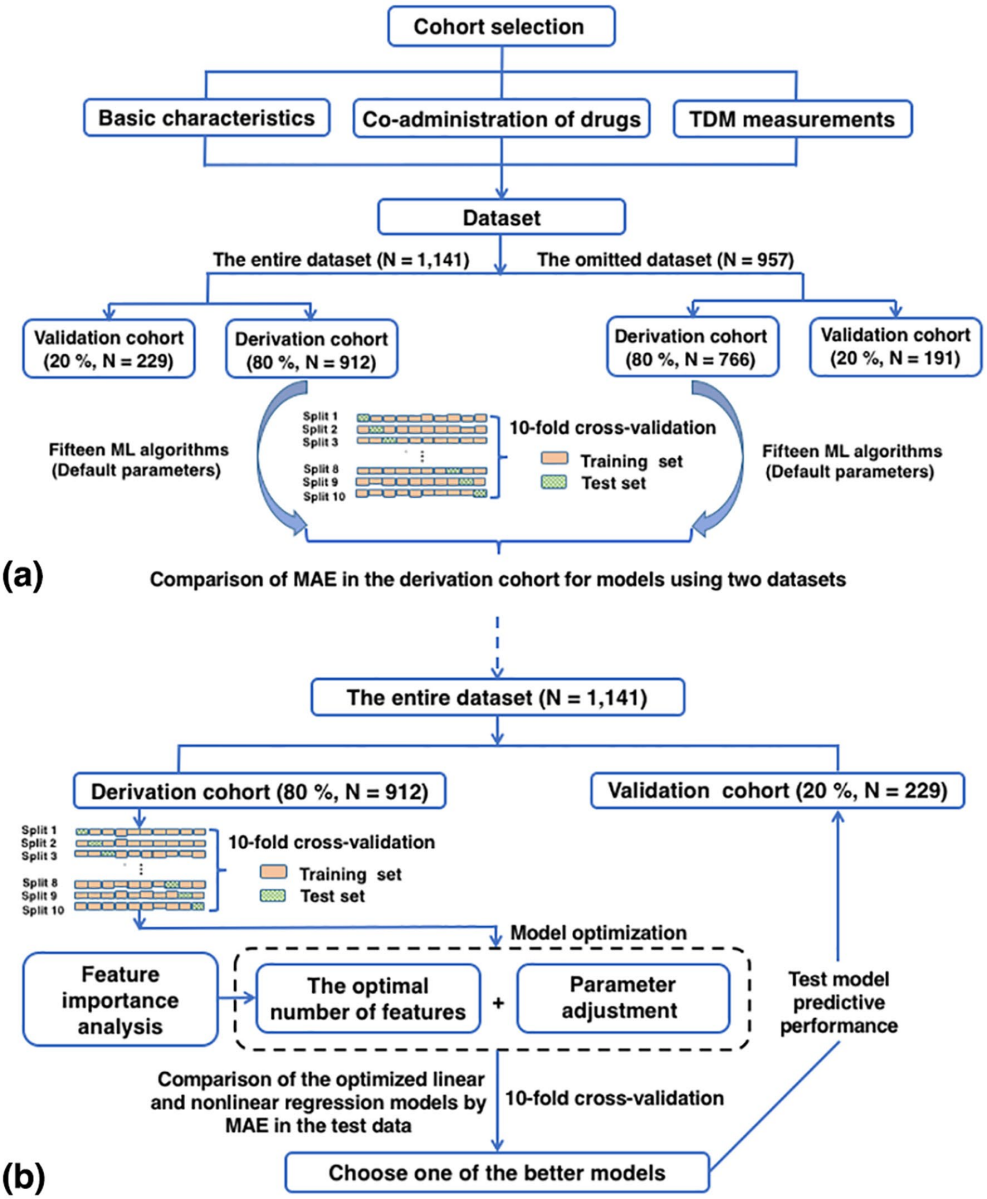
Flowchart of data collection, analysis of missing data, model selection, and model validation. (**a**) Comparisons of models in terms of the MAE on the test data using the entire dataset and the omitted dataset. (**b**) Comparisons of the performance of linear and nonlinear models in terms of MAE on the test data using the entire dataset. *MAE* mean absolute error, *TDM* therapeutic drug-monitoring, *ML* machine learning.

We selected clinical factors related to the C/D ratio of LTG, and built a prediction model to adjust its doses and minimize adverse reactions. Our ML process can be divided into five steps: (1) data preprocessing, (2) imputation evaluation, (3) model optimization, (4) model selection, and (5) model validation. The overall modeling process is illustrated in Fig. 5.

#### Data preprocessing

The process of data cleaning and transformation is intended to solve problems of missing values and data normalization. Before analysis, summary and descriptive statistics were obtained, with a review of the missing values. If the rate of absence of a variable in the original dataset was above 50%, it was removed^51^. The missing values needed to be computed before the data were input into the model. We filled in the missing values by imputation using the k-nearest neighbor (KNN) method with *k* = 3^51^. Data normalization was also performed to obtain data of higher quality. Categorical variables were coded using “one-hot” encoding, and continuous variables were rescaled using min–max rescaling^52^.

#### Imputation evaluation

The imputation evaluation was based on two datasets: the entire dataset (N = 1141) with missing data points imputed, and the omitted dataset (N = 957) derived from the entire dataset but with the missing data points omitted. For each dataset, the raw data were randomly split into two batches. Eighty percent of the raw data were randomly selected as the "derivation cohort", and the remaining were assigned to the "validation cohort." To explore whether imputation generated a bias, we employed tenfold cross-validation to compare the performance of models using the two datasets above^53^: the entire derivation cohort was divided into 10 subsets, and the holdout method was repeated 10 times. Each of the subsets was used in turn as the test set and the other nine subsets were combined to form a training set (see Fig. 5). The average MAE across all 10 tests was then computed. All features in the dataset were considered, and the hyperparameters for each algorithm used in these comparisons were set to their default values specified in the package^54^. They are listed in Supplementary Table S17.

*R*^2^, MAE, mean square error (MSE), and root mean square error (RMSE) are commonly used evaluation metrics for regression models in ML. Measures of dispersion, such as the MAE and RMSE, are preferred over *R*^2^ because the value of a model generally lies in its overall accuracy and precision, but not on how successfully it explains the dependent data variance^55^. To explore the impact of imputation on prediction, that is, the differences in MAE in the test set before and after omitting the missing data points, paired sample t-tests were performed. Defined as the average of the absolute values of the predicted C/D ratio ($y_{i}$) minus the observed C/D ratio ($\hat{y}_{i}$), the MAE was calculated according to Eq. (1):

1 $${\text{MAE }}\left( {y,\hat{y}} \right){ = }\frac{{1}}{{n_{{{\text{samples}}}} }}\mathop \sum \limits_{i = 1}^{{n_{{{\text{samples}}}} }} \left| {y_{i} - \hat{y}_{i} } \right|$$

#### Model optimization

Model optimization involved feature selection and parameter adjustment. Before this step, raw data from the entire dataset were randomly split into two batches. Eighty percent (912 measurements) of the TDM measurements were randomly selected as the "derivation cohort" to develop an optimal regression model. The remaining 20% (229 measurements) were assigned to the "validation cohort," which was used to test the performance of the final model in terms of handling unseen samples. Only the derivation cohort was used for feature selection and parameter adjustment. Feature selection, which is crucial for developing a better and simpler model, is the process of selecting a subset of relevant variables (features) to be used for regression by removing redundant and/or irrelevant ones^56^. Here, an embedded approach to feature selection was chosen during training, together with a suitable regression algorithm, such as the algorithm evaluated here or the random forest regression algorithm, if the evaluated algorithm had no attribute with “feature_importance_.” Features that best contributed to the model were learned while the model was being created^56^. They were then ranked according to measured importance. However, an optimal feature set should have had the fewest variables but the best predictive performance. Therefore, a forward feature selection strategy was used to find the shortest list of features. Briefly, forward feature selection began at the top of a ranked list of features, and iteratively generated increasingly longer lists, along with the corresponding models and their predictive performances on the test set using tenfold cross-validation by adding one feature at a time^57^. The C/D ratio and the corresponding feature covariates selected above were evaluated via Pearson’s correlation visualization and heatmap analysis.

Once the optimal number of features had been determined, parameter adjustment was needed to optimize the learning algorithm. In general, we did this using tenfold cross-validation by choosing important hyperparameters first. The optimal parameters were filtered by mean cross-validation scores on the test set. As in the forward feature selection strategy, the parameter adjustment strategy involved iteratively generating increasingly larger parameter values and their corresponding scores. Finally, tenfold cross-validation was used in a grid search of the other key tuning parameters.

#### Model selection

As in feature selection and parameter adjustment, only the derivation cohort in the entire dataset was used for model selection. A total of 15 supervised ML algorithms were applied and evaluated: AdaBoost regression (ABR), bagging regression (BR), decision tree regression (DTR), extra-trees’ regression (ETR), gradient-boosted regression (GBR), k-nearest neighbor regression (KNR), lasso regression (LassoR), multiple linear regression (MLR), linear support vector regression (LSVR), multi-layer perceptron regression (MLPR), nu-support vector regression (NuSVR), random forest regression (RFR), ridge regression (RidgeR), support vector regression (SVR), and XGBoost regression (XGBR). The hyperparameters of each algorithm were optimized and the corresponding optimal feature set was determined according to the methods of feature selection and parameter adjustment considered. Considering the small size of our dataset, and to avoid bias, we employed tenfold cross-validation to compare the performance of the models^53^. They were then filtered by the MAE in the test data. To test the differences in the predictive performance of the models, unpaired student’s t-tests were performed. One of the better models was then selected for validation, and its performance before and after optimization was compared using a paired sample t-test.

All models were developed and evaluated in Python (version 3.7.2) using Anaconda (version 5.3.1, https://www.anaconda.com/; Anaconda Inc., Austin, TX, USA), Jupyter notebook (version 5.6.0, https://jupyter.org), the scikit-learn package (version 0.20.2, http://scikit-learn.org/stable/), and the XGBoost package (version 0.80, https://xgboost.readthedocs.io/en/latest/). All statistical analyses and data visualizations were implemented using Python (version 3.7.2) with the relevant packages: the scipy package (version 1.2.1, https://www.scipy.org), the matplotlib package (version 2.3.2, https://matplotlib.org), and the seaborn package (version 0.9.0, http://seaborn.pydata.org).

#### Model validation

The shortest list of features and the most suitable parameters were determined for the chosen algorithm to yield a better predictive model. Model validation was performed using three indices: MAE, MRE (%), and the percentage of predictions within 20% of the observed C/D ratios (percentage within 20%) in the validating cohort. The MRE (%), defined as the average of the ratio of an error in the predicted C/D ratio ($y_{i}$) to the magnitude of the observed C/D ratio ($\hat{y}_{i}$), was calculated according to Eq. (2):

2 $${\text{MRE}} \left( \% \right) = \frac{1}{{n_{{{\text{samples}}}} }}\mathop \sum \limits_{i = 1}^{{n_{{{\text{samples}}}} }} \frac{{\left( {y_{i} - \hat{y}_{i} } \right)}}{{\hat{y}_{i} }} \times 100\%$$

We selected the percentage within 20% because this range has been widely accepted and applied to assess models for predicting the dosages of such drugs as warfarin and tacrolimus (see Table 1)^19–22^. Moreover, ignoring problems of adherence and pharmacokinetic alteration, the intra-individual variation in the C/D ratio is generally considered to not be above 20%^7^. The above indices were calculated overall for the validation cohort, and in terms of the range of the C/D ratio based on the 25% quartile of the unseen data points, where this was divided into two categories: low C/D ratio range (< 22.1 μg mL^−1^ g^−1^ day) and intermediate-to-high C/D ratio range (≥ 22.1 μg mL^−1^ g^−1^ day). Given the wide range of values of the C/D ratio, we also considered modeling log_10_(C/D ratio) when treated as a label by using the proposed algorithm. In the context of modeling the C/D ratio and log_10_(C/D ratio), the respective performances of the optimized models on the validation cohort based on different ranges of the C/D ratio were compared.

## Supplementary Information

Supplementary Information
