# Supplementary material for: A machine learning approach to personalized dose adjustment of lamotrigine using noninvasive clinical parameters
Source: Sci Rep. 2021 Mar 10;11:5568. doi: 10.1038/s41598-021-85157-x (PMC7946912; doi:10.1038/s41598-021-85157-x)
Supplement: Supplementary file 1 — Supplementary Information [file 41598_2021_85157_MOESM1_ESM.pdf]

Title:

A Machine Learning Approach to Personalized Dose Adjustment of Lamotrigine Using Noninvasive Clinical Parameters

Authors:

Xiuqing Zhu[1,2], Wencan Huang[3], Haoyang Lu[1,2], Zhanzhang Wang[1,2], Xiaojia Ni[1,2], Jinqing Hu[1,2], Shuhua Deng[1,2], Yaqian Tan[1,2], Lu Li[1,2], Ming Zhang[1,2], Chang Qiu[1,2], Yayan Luo[2,4], Hongzhen Chen[1], Shanqing Huang[1], Tao Xiao[1], Dewei Shang[1,2,*], and Yuguan Wen[1,2,*]

Authors affiliations:

[1] Department of Pharmacy, The Affiliated Brain Hospital of Guangzhou Medical University (Guangzhou Huiai Hospital), Guangzhou, 510370, China.

[2] Guangdong Engineering Technology Research Center for Translational Medicine of Mental Disorders, Guangzhou, 510370, China.

[3] Department of Pharmacy, Guangzhou Bureau of Civil Affairs Psychiatric Hospital, Guangzhou, 510430, China.

[4] Institute of Neuropsychiatry, The Affiliated Brain Hospital of Guangzhou Medical University (Guangzhou Huiai Hospital), Guangzhou, 510370, China.

*** Corresponding Authors:**

Dewei Shang,

Department of Pharmacy, The Affiliated Brain Hospital of Guangzhou Medical University (Guangzhou Huiai Hospital), Guangzhou, 510370, China.

Tel.: +86-02081268389

Fax: +86-02081891391

E-mail: shang_dewei@163.com

Yuguan Wen,

Department of Pharmacy, The Affiliated Brain Hospital of Guangzhou Medical University (Guangzhou Huiai Hospital), Guangzhou, 510370, China.

Tel.: +86-02081268389

Fax: +86-02081891391

E-mail: wenyuguandede@163.com

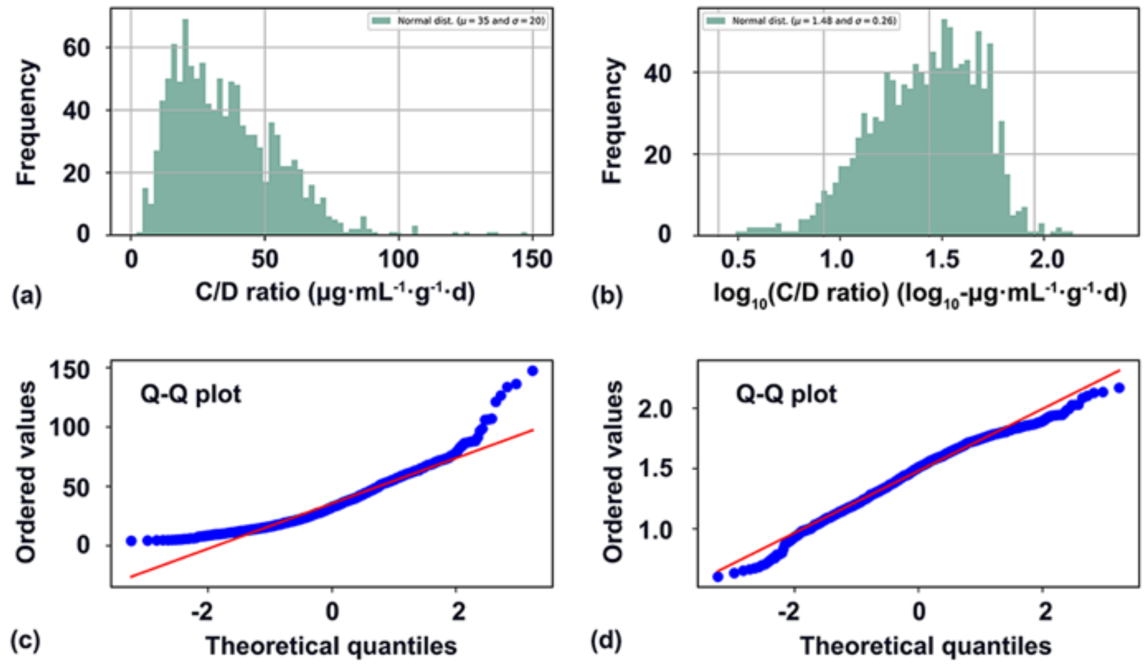

Figure S1 [Panel (**a**)]: Histogram of the dose-adjusted concentrations (C/D ratio). [Panel (**b**)]: Histogram of $\log_{10}$(C/D ratio). [Panel (**c**)]: Quantile–quantile (Q-Q) plot of the C/D ratio. [Panel (**d**)]: Q-Q plot of $\log_{10}$(C/D ratio).

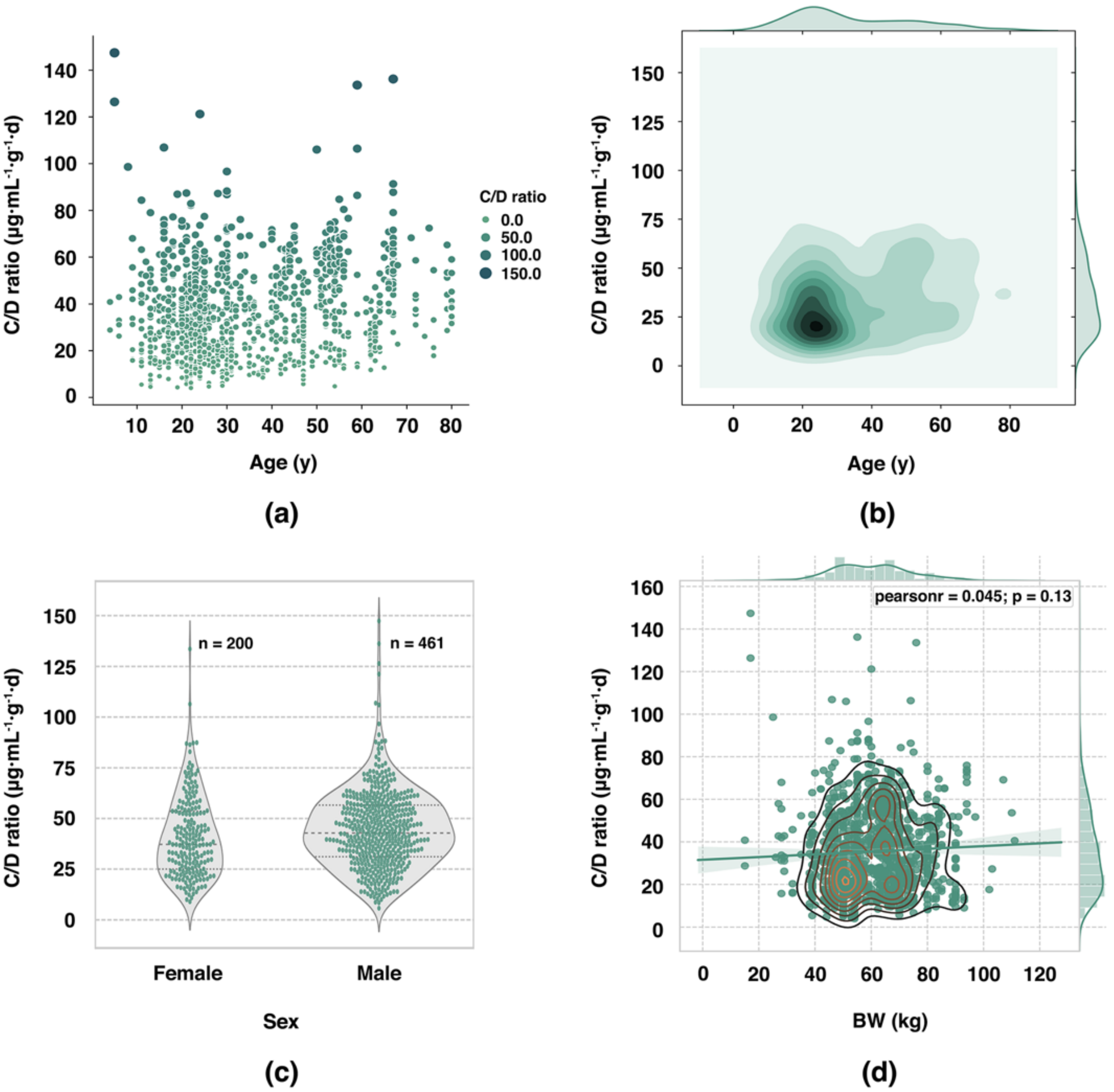

Figure S2 [Panel (**a**)]: Scatter plot of age vs. observed dose-adjusted concentrations (C/D ratio). [Panel (**b**)]: Kernel density estimation (KDE) plot of age vs. C/D ratio. [Panel (**c**)]: Violin plot of sex vs. C/D ratio in the context of the co-administration of valproic acid. [Panel (**d**)]: Scatter plot and KDE plot of body weight (BW) vs. C/D ratio.

Table S1 Grid search-based parameter optimization for extra-trees' regression algorithm using 10-fold cross-validation in the context of modeling the C/D ratio.

Steps*	Parameters tuned	Grid Search Script	Optimal performance
Step 1	n_estimators<br>(1,2,3,4,5,6,7,8,9,10,11,12,13,14,15,<br>16,17,18,19,20,21,22,23,24,25,26,27,<br>28,29,30,31,32,33,34,35,36,37,38,39,<br>40,41,42,43,44,45,46,47,48,49,50)	grid_params = {'n_estimators':np.arange<br>(1,51)}<br>rf = ExtraTreesRegressor(random_state=0)<br>grid = GridSearchCV(rf, grid_params, cv = 10)	0.5093<br>n_estimators=31
Step 2	max_depth<br>(10,11,12,13,14,15,16,17,18,19,20,21,<br>22,23,24)	grid_params = {'max_depth':np.arange (10,25)}<br>rf = ExtraTreesRegressor(n_estimators=31,<br>random_state=0)<br>grid = GridSearchCV(rf, grid_params, cv = 10)	0.5364<br>max_depth=12
Step 3	min_samples_leaf<br>(1,2,3,4,5,6,7,8,9,10)<br>min_samples_split (2,3,4,5,6)	grid_params =<br>{'min_samples_leaf':np.arange(1,11),<br>'min_samples_split':np.arange(2,7)}<br>rf = ExtraTreesRegressor(n_estimators=31,<br>max_depth = 12, random_state=0)<br>grid = GridSearchCV(rf,grid_params,cv = 10)	0.5486<br>min_samples_leaf=1<br>min_samples_split=3
Step 4	max_features ('auto', 'sqrt')	grid_params = {'max_features':['auto', 'sqrt']}<br>rf = ExtraTreesRegressor(n_estimators=31,<br>max_depth = 12, min_samples_leaf=1,<br>min_samples_split=3, random_state=0)<br>grid = GridSearchCV(rf,grid_params,cv = 10)	0.5486<br>max_features='auto'
Step 5	max_depth<br>(10,11,12,13,14,15,16,17,18,19,20,21,<br>22,23,24)<br>min_samples_leaf<br>(1,2,3,4,5,6,7,8,9,10)<br>min_samples_split (2,3,4,5,6)<br>max_features ('auto', 'sqrt')	grid_params = {'max_depth':np.arange(10,25),<br>'min_samples_leaf':np.arange(1,11),<br>'min_samples_split':np.arange(2,7),<br>'max_features':['auto', 'sqrt']}<br>rf = ExtraTreesRegressor(n_estimators=31,<br>random_state=0)<br>grid = GridSearchCV(rf,grid_params,cv = 10)	0.5510<br>max_depth=20<br>min_samples_leaf=1<br>min_samples_split=6<br>max_features='auto'

* The optimal feature set [including daily dosage of valproic acid, age, body weight, enzyme inducers, and sex (male)] was determined before parameter adjustment.

Table S2 Grid search-based parameter optimization for k-nearest neighbor regression algorithm using 10-fold cross-validation in the context of modeling the C/D ratio.

Steps*	Parameters tuned	Grid Search Script	Optimal performance
Step 1	weights ('uniform', 'distance')<br>n_neighbors (1,2,3,4,5,6,7,8,9,10)<br>p (1,2,3,4,5)	grid_params = [{'weights': ['uniform'],<br>'n_neighbors': [1,2,3,4,5,6,7,8,9,10]},<br>{'weights': ['distance'],<br>'n_neighbors': [1,2,3,4,5,6,7,8,9,10],<br>'p': [1,2,3,4,5]}]<br>rf = KNeighborsRegressor()<br>grid = GridSearchCV(rf,grid_params,cv = 10)	0.5125<br>weights='distance'<br>n_neighbors=9<br>p=1

* The optimal feature set [including daily dosage of valproic acid, body weight, age, enzyme inducers, and sex (male)] was determined before parameter adjustment.

Table S3 Grid search-based parameter optimization for bagging regression algorithm using 10-fold cross-validation in the context of modeling the C/D ratio.

Steps*	Parameters tuned	Grid Search Script	Optimal performance
Step 1	n_estimators (1,2,3,4,5,6,7,8,9,10,11,12,13,14,15, 16,17,18,19,20,21,22,23,24,25,26,27, 28,29,30,31,32,33,34,35,36,37,38,39, 40,41,42,43,44,45,46,47,48,49,50,51, 52,53,54,55,56,57,58,59,60,61,62,63, 64,65,66,67,68,69,70,71,72,73,74,75, 76,77,78,79,80,81,82,83,84,85,86,87, 88,89,90,91,92,93,94,95,96,97,98,99, 100)	grid_params = {'n_estimators':np.arange (1,101)}<br>rf = BaggingRegressor(random_state=0)<br>grid = GridSearchCV(rf, grid_params, cv = 10)	0.5250<br>n_estimators=82
Step 2	max_features (0.05, 0.1, 0.2, 0.5, 0.6, 0.7, 0.75, 0.8, 0.85, 0.9, 0.95, 1.0)<br>max_samples (0.05, 0.1, 0.2, 0.5, 0.6, 0.7, 0.75, 0.8, 0.85, 0.9, 0.95, 1.0)	grid_params = {'max_features': [0.05, 0.1, 0.2, 0.5, 0.6, 0.7, 0.75, 0.8, 0.85, 0.9, 0.95, 1.0], 'max_samples': [0.05, 0.1, 0.2, 0.5, 0.6, 0.7, 0.75, 0.8, 0.85, 0.9, 0.95, 1.0]}<br>rf = BaggingRegressor(n_estimators=82)<br>grid = GridSearchCV(rf, grid_params, cv = 10)	0.5465<br>max_features=0.85<br>max_samples=0.95

* The optimal feature set (including daily dosage of valproic acid, age, body weight, and enzyme inducers) was determined before parameter adjustment.

Table S4 Grid search-based parameter optimization for random forest regression algorithm using 10-fold cross-validation in the context of modeling the C/D ratio.

Steps*	Parameters tuned	Grid Search Script	Optimal performance
Step 1	n_estimators (1,2,3,4,5,6,7,8,9,10,11,12,13,14,15, 16,17,18,19,20,21,22,23,24,25,26,27, 28,29,30,31,32,33,34,35,36,37,38,39, 40,41,42,43,44,45,46,47,48,49,50,51, 52,53,54,55,56,57,58,59,60,61,62,63, 64,65,66,67,68,69,70,71,72,73,74,75, 76,77,78,79,80,81,82,83,84,85,86,87, 88,89,90,91,92,93,94,95,96,97,98,99, 100)	grid_params = {'n_estimators':np.arange(1,101)}<br>rf = RandomForestRegressor(random_state=0)<br>grid = GridSearchCV(rf, grid_params, cv = 10)	0.5343<br>n_estimators=83
Step 2	max_depth (3,4,5,6,7,8,9,10,11,12,13,14)<br>min_samples_split (2,3,4,5,6,7,8,9,10,11,12,13,14)<br>min_samples_leaf (1,2)	grid_params = {'max_depth': range(3,15,1), 'min_samples_split': range(2,15,1), 'min_samples_leaf': range(1,3,1) }<br>rf = RandomForestRegressor(n_estimators=83)<br>grid = GridSearchCV(rf,grid_params, cv = 10)	0.5467<br>max_depth=11<br>min_samples_split=4<br>min_samples_leaf =1

* The optimal feature set [including daily dosage of valproic acid, age, body weight, enzyme inducers, and sex (male)] was determined before parameter adjustment.

Table S5 Grid search-based parameter optimization for XGBoost regression algorithm using 10-fold cross-validation in the context of modeling the C/D ratio.

Steps*	Parameters tuned	Grid Search Script	Optimal performance
Step 1	n_estimators (100,101,102,103,104,105,106,107, 108,109,110,111,112,113,114,115, 116,117,118,119,120,121,122,123, 124,125,126,127,128,129,130,131, 132,133,134,135,136,137,138,139, 140,141,142,143,144,145,146,147, 148,149,150,151,152,153,154,155, 156,157,158,159,160)	grid_params = {'n_estimators' : np.arange(100,161)}<br>rf = XGBRegressor(random_state=0)<br>grid = GridSearchCV(rf,grid_params,cv = 10)	0.5405<br>n_estimators=151
Step 2	max_depth (1,2,3,4,5,6,7,8,9)<br>min_child_weight (1,2,3,4,5)	grid_params = {'max_depth': range(1,10,1), 'min_child_weight': range(1,6,1) }<br>rf = XGBRegressor(n_estimators=151)<br>grid = GridSearchCV(rf,grid_params, cv = 10)	0.5405<br>max_depth=3<br>min_child_weight=1
Step 3	gamma<br>(0, 0.1, 0.2, 0.3, 0.4 , 0.5, 0.6, 0.7, 0.8, 0.9)	grid_params = {'gamma': [0, 0.1, 0.2, 0.3, 0.4, 0.5, 0.6, 0.7, 0.8, 0.9]}<br>rf = XGBRegressor(n_estimators=151, max_depth=3, min_child_weight=1)<br>grid = GridSearchCV(rf, grid_params, cv = 10)	0.5405<br>gamma=0
Step 4	subsample<br>(0.5, 0.6, 0.7, 0.8, 0.9, 1.0)<br>colsample_bytree<br>(0.5, 0.6, 0.7, 0.8, 0.9, 1.0)	grid_params = {'subsample': [0.5, 0.6, 0.7, 0.8, 0.9, 1.0], 'colsample_bytree': [0.5, 0.6, 0.7, 0.8, 0.9, 1.0]}<br>rf = XGBRegressor(n_estimators=151, max_depth=3, min_child_weight=1, gamma=0)<br>grid = GridSearchCV(rf, grid_params, cv = 10)	0.5439<br>colsample_bytree= 0.5<br>subsample=1.0
Step 5	reg_alpha<br>(0, 0.05, 0.1, 1, 2, 3, 4, 5, 6)<br>reg_lambda (0.05, 0.1, 1, 2, 3, 4, 5, 6)	grid_params = {'reg_alpha': [0, 0.05, 0.1, 1, 2, 3, 4, 5, 6], 'reg_lambda': [0.05, 0.1, 1, 2, 3, 4, 5, 6]}<br>rf = XGBRegressor(n_estimators=151, max_depth=3, min_child_weight=1, gamma=0, colsample_bytree=0.5, subsample=1.0)<br>grid = GridSearchCV(rf, grid_params, cv = 10)	0.5450<br>reg_alpha=4,<br>reg_lambda=2
Step 6	learning_rate<br>(0, 0.05, 0.1, 0.2, 0.3, 0.4, 0.5, 0.6, 0.7, 0.8, 0.9, 1)	grid_params = {'learning_rate': [0, 0.05, 0.1, 0.2, 0.3, 0.4, 0.5, 0.6, 0.7, 0.8, 0.9, 1]}<br>rf = XGBRegressor(n_estimators=151, max_depth=3, min_child_weight=1, gamma=0, colsample_bytree=0.5, subsample=1.0, reg_alpha=4, reg_lambda=2)<br>grid = GridSearchCV(rf, grid_params, cv = 10)	0.5450<br>learning_rate=0.1

* The optimal feature set [including enzyme inducers, daily dosage of valproic acid, daily dosage of carbamazepine, age, daily dosage of phenobarbitone, body weight, sex (male), and daily dosage of oxcarbazepine] was determined before parameter adjustment.

Table S6 Grid search-based parameter optimization for gradient boosted regression algorithm using 10-fold cross-validation in the context of modeling the C/D ratio.

Steps*	Parameters tuned	Grid Search Script	Optimal performance
Step 1	n_estimators (100,101,102,103,104,105,106,107, 108,109,110,111,112,113,114,115, 116,117,118,119,120,121,122,123, 124,125,126,127,128,129,130,131, 132,133,134,135,136,137,138,139, 140,141,142,143,144,145,146,147, 148,149,150)	grid_params = {'n_estimators' :np.arange(100,151)}<br>rf = GradientBoostingRegressor(random_state=0)<br>grid = GridSearchCV(rf,grid_params,cv = 10)	0.5330<br>n_estimators=134
Step 2	max_depth (3,4,5,6,7,8,9,10,11,12,13,14)<br>min_samples_split (2,3,4,5,6,7,8,9,10,11,12,13,14)<br>min_samples_leaf (1,2)	grid_params = {'max_depth': range(3,15,1), 'min_samples_split': range(2,15,1), 'min_samples_leaf': range(1,3,1) }<br>rf = GradientBoostingRegressor(n_estimators=134)<br>grid = GridSearchCV(rf,grid_params, cv = 10)	0.5394<br>max_depth=3<br>min_samples_split=5<br>min_samples_leaf =1

* The optimal feature set [including daily dosage of valproic acid, age, body weight, enzyme inducers, sex (male), and daily dosage of oxcarbazepine] was determined before parameter adjustment.

Table S7 Grid search-based parameter optimization for decision tree regression algorithm using 10-fold cross-validation in the context of modeling the C/D ratio.

Steps*	Parameters tuned	Grid Search Script	Optimal performance
Step 1	criterion ('mse', 'mae')	grid_params = {'criterion': ('mse', 'mae')}<br>rf = DecisionTreeRegressor(random_state=0)<br>grid = GridSearchCV(rf,grid_params,cv = 10)	0.3595<br>criterion='mse'
Step 2	max_depth (1,2,3,4,5,6,7,8,9,10,11,12,13,14)<br>min_samples_split (2,3,4,5,6,7)<br>min_samples_leaf (1,2,3,4,5,6,7)	grid_params = {'max_depth':np.arange(1, 15), 'min_samples_split':np.arange(2,8), 'min_samples_leaf':np.arange(1,8)}<br>rf = DecisionTreeRegressor(criterion='mse', random_state=0)<br>grid = GridSearchCV(rf,grid_params,cv = 10)	0.5011<br>max_depth=11<br>min_samples_split=2<br>min_samples_leaf =2

* The optimal feature set (including daily dosage of valproic acid, body weight, age, and enzyme inducers) was determined before parameter adjustment.

Table S8 Grid search-based parameter optimization for support vector regression algorithm using 10-fold cross-validation in the context of modeling the C/D ratio.

Steps*	Parameters tuned	Grid Search Script	Optimal performance
Step 1	C (100, 150, 200, 250, 300, 350, 400)<br>gamma (2, 4, 8, 10, 12, 14, 16)<br>epsilon (0.1, 1.0, 2.0, 3.0, 4.0)	grid_params = {'C': [100, 150, 200, 250, 300, 350, 400],<br>'gamma': [2, 4, 8, 10, 12, 14, 16],<br>'epsilon': [0.1, 1.0, 2.0, 3.0, 4.0]}<br>rf = SVR()<br>grid = GridSearchCV(rf, grid_params, cv = 10)	0.4511<br>C=300<br>gamma=14<br>epsilon=1.0

* The optimal feature set [including daily dosage of valproic acid, body weight, age, enzyme inducers, sex (male), and daily dosage of oxcarbazepine] was determined before parameter adjustment.

Table S9 Grid search-based parameter optimization for nu-support vector regression algorithm using 10-fold cross-validation in the context of modeling the C/D ratio.

Steps*	Parameters tuned	Grid Search Script	Optimal performance
Step 1	C (200, 300, 400, 500, 600)<br>gamma (5, 8, 10, 12, 14, 16, 18)<br>nu (0.1, 0.3, 0.5, 0.7, 1.0)	grid_params = {'C': [200, 300, 400, 500, 600],<br>'gamma': [5, 8, 10, 12, 14, 16, 18],<br>'nu': [0.1, 0.3, 0.5, 0.7, 1.0]}<br>rf = NuSVR()<br>grid = GridSearchCV(rf, grid_params, cv = 10)	0.4508<br>C=500<br>gamma=10<br>nu=0.7

* The optimal feature set [including daily dosage of valproic acid, body weight, age, enzyme inducers, sex (male), and daily dosage of oxcarbazepine] was determined before parameter adjustment.

Table S10 Grid search-based parameter optimization for multi-layer perceptron regression algorithm using 10-fold cross-validation in the context of modeling the C/D ratio.

Steps*	Parameters tuned	Grid Search Script	Optimal performance
Step 1	hidden_layer_sizes<br>((100,), (100,100), (100,100,100),<br>(100,100,100,100))	grid_params = {'hidden_layer_sizes':<br>((100,), (100,100), (100,100,100),<br>(100,100,100,100))}<br>rf = MLPRegressor(random_state=0)<br>grid = GridSearchCV(rf,grid_params,cv = 10)	0.4303<br>hidden_layer_sizes=<br>(100, 100, 100, 100)
Step 2	activation<br>('identity','logistic','tanh','relu')	grid_params =<br>{'activation':('identity','logistic','tanh','relu')}<br>rf = MLPRegressor(hidden_layer_sizes=<br>(100, 100,100,100), random_state=0)<br>grid = GridSearchCV(rf,grid_params,cv = 10)	0.4303<br>activation='relu'
Step 3	alpha<br>(0.0001, 0.001, 0.01, 0.1, 1)	grid_params = {'alpha':<br>[0.0001,0.001,0.01,0.1,1]}<br>rf = MLPRegressor(hidden_layer_sizes=(100,<br>100,100,100), activation='relu',<br>random_state=0)<br>grid = GridSearchCV(rf,grid_params, cv = 10)	0.4376<br>alpha=0.1

* The optimal feature set [including daily dosage of valproic acid, age, body weight, enzyme inducers, and sex (male)] was determined before parameter adjustment.

Table S11 Grid search-based parameter optimization for AdaBoost regression algorithm using 10-fold cross-validation in the context of modeling the C/D ratio.

Steps*	Parameters tuned	Grid Search Script	Optimal performance
Step 1	n_estimators<br>(1,2,3,4,5,6,7,8,9,10,11,12,13,14,15,<br>16,17,18,19,20,21,22,23,24,25,26,27,<br>28,29,30,31,32,33,34,35,36,37,38,39,<br>40,41,42,43,44,45,46,47,48,49,50)<br>loss ('linear', 'square', 'exponential')<br>learning_rate (0.001, 0.01, 0.1, 1.0)	grid_params = {'n_estimators': np.arange(1,51),<br>'loss': ['linear', 'square', 'exponential'],<br>'learning_rate': [0.001, 0.01, 0.1, 1.0]}<br>rf = AdaBoostRegressor(random_state=0)<br>grid = GridSearchCV(rf,grid_params,cv = 10)	0.3183<br>n_estimators=15<br>loss='exponential'<br>learning_rate=0.001

* The optimal feature set (including daily dosage of valproic acid) was determined before parameter adjustment.

Table S12 Grid search-based parameter optimization for multiple linear regression algorithm using 10-fold cross-validation in the context of modeling the C/D ratio.

Steps*	Parameters tuned	Grid Search Script	Optimal performance
Step 1	fit_intercept ('True', 'False')<br>normalize ('True', 'False')<br>n_jobs (-1,0,1,2,3)<br>copy_X ('True', 'False')	grid_params = {'fit_intercept': ['True','False'],<br>'normalize': ['True','False'],<br>'n_jobs': [-1,0,1,2,3],<br>'copy_X': ['True','False']}<br>rf = LinearRegression()<br>grid = GridSearchCV(rf,grid_params,cv = 10)	0.1921<br>fit_intercept ='True'<br>normalize ='True'<br>n_jobs=-1<br>copy_X='True'

* The optimal feature set [including daily dosage of valproic acid, age, body weight, enzyme inducers, sex (male), and daily dosage of oxcarbazepine] was determined before parameter adjustment.

Table S13 Grid search-based parameter optimization for ridge regression algorithm using 10-fold cross-validation in the context of modeling the C/D ratio.

Steps*	Parameters tuned	Grid Search Script	Optimal performance
Step 1	alpha<br>(0.0001, 0.001, 0.01, 0.1, 1, 10, 100)	grid_params = {'alpha': [0.0001, 0.001, 0.01, 0.1, 1, 10, 100]}<br>rf = Ridge()<br>grid = GridSearchCV(rf,grid_params,cv = 10)	0.1937<br>alpha=1
Step 2	alpha<br>(0.5, 0.7, 0.9, 1, 1.2, 1.5, 1.7, 2, 5, 7, 10)	grid_params = {'alpha': [0.5, 0.7, 0.9, 1, 1.2, 1.5, 1.7, 2, 5, 7, 10]}<br>rf = Ridge()<br>grid = GridSearchCV(rf, grid_params, cv = 10)	0.1940<br>alpha=1.7

* The optimal feature set [including daily dosage of valproic acid, age, body weight, enzyme inducers, sex (male), and daily dosage of oxcarbazepine] was determined before parameter adjustment.

Table S14 Grid search-based parameter optimization for lasso regression algorithm using 10-fold cross-validation in the context of modeling the C/D ratio.

Steps*	Parameters tuned	Grid Search Script	Optimal performance
Step 1	alpha<br>(0.0001, 0.001, 0.01, 0.1, 1, 10, 100)	grid_params = {'alpha': [0.0001, 0.001, 0.01, 0.1, 1, 10, 100]}<br>rf = Lasso()<br>grid = GridSearchCV(rf,grid_params,cv = 10)	0.1922<br>alpha=0.01
Step 2	alpha<br>(0.005, 0.008, 0.01, 0.015, 0.02)	grid_params = {'alpha': [0.005, 0.008, 0.01, 0.015, 0.02]}<br>rf = Lasso()<br>grid = GridSearchCV(rf,grid_params,cv = 10)	0.1923<br>alpha=0.015
Step 3	alpha<br>(0.01, 0.012, 0.015, 0.017, 0.02)	grid_params = {'alpha': [0.01, 0.012, 0.015, 0.017, 0.02]}<br>rf = Lasso()<br>grid = GridSearchCV(rf,grid_params,cv = 10)	0.1923<br>alpha=0.017

* The optimal feature set [including daily dosage of valproic acid, age, body weight, enzyme inducers, sex (male), and daily dosage of oxcarbazepine] was determined before parameter adjustment.

Table S15 Grid search-based parameter optimization for linear-support vector regression algorithm using 10-fold cross-validation in the context of modeling the C/D ratio.

Steps*	Parameters tuned	Grid Search Script	Optimal performance
Step 1	C<br>(50, 60, 70, 75, 80, 83, 85, 88, 90, 92, 95, 98, 100)<br>loss<br>('epsilon_insensitive', 'squared_epsilon_insensitive')<br>epsilon<br>(1.0, 2.0, 2.5, 2.7, 2.8, 2.9, 3.0, 3.1, 3.2, 3.3, 3.4, 3.5, 4.0)	grid_params = {'C': [50, 60, 70, 75, 80, 83, 85, 88, 90, 92, 95, 98, 100],<br>'loss': ['epsilon_insensitive', 'squared_epsilon_insensitive'],<br>'epsilon': [1.0, 2.0, 2.5, 2.7, 2.8, 2.9, 3.0, 3.1, 3.2, 3.3, 3.4, 3.5, 4.0]}<br>rf = LinearSVR()<br>grid = GridSearchCV(rf, grid_params, cv = 10)	0.1974<br>C=88<br>loss= 'squared_epsilon_insensitive'<br>epsilon=2.9

* The optimal feature set [including daily dosage of valproic acid, age, body weight, enzyme inducers, sex (male), and daily dosage of oxcarbazepine] was determined before parameter adjustment.

Table S16 Grid search-based parameter optimization for extra-trees' regression algorithm using 10-fold cross-validation in the context of modeling $\log_{10}$(C/D ratio).

Steps*	Parameters tuned	Grid Search Script	Optimal performance
Step 1	n_estimators (1,2,3,4,5,6,7,8,9,10,11,12,13,14,15, 16,17,18,19,20,21,22,23,24,25,26,27, 28,29,30,31,32,33,34,35,36,37,38,39, 40,41,42,43,44,45,46,47,48,49,50)	grid_params = {'n_estimators':np.arange (1,51)}<br>rf = ExtraTreesRegressor(random_state=0)<br>grid = GridSearchCV(rf, grid_params, cv = 10)	0.5430<br>n_estimators=34
Step 2	max_depth (1,2,3,4,5,6,7,8,9,10,11,12,13,14)	grid_params = {'max_depth':np.arange (1,15)}<br>rf = ExtraTreesRegressor(n_estimators=34, random_state=0)<br>grid = GridSearchCV(rf, grid_params, cv = 10)	0.5714<br>max_depth=12
Step 3	min_samples_leaf (1,2,3,4,5,6,7,8,9,10)<br>min_samples_split (2,3,4,5,6,7,8,9,10)	grid_params = {'min_samples_leaf':np.arange(1,11), 'min_samples_split':np.arange(2,11)}<br>rf = ExtraTreesRegressor(n_estimators=34, max_depth = 12, random_state=0)<br>grid = GridSearchCV(rf,grid_params,cv = 10)	0.5749<br>min_samples_leaf=2<br>min_samples_split=2
Step 4	max_features ('auto', 'sqrt')	grid_params = {'max_features':['auto', 'sqrt']}<br>rf = ExtraTreesRegressor(n_estimators=34, max_depth = 12, min_samples_leaf=2, min_samples_split=2, random_state=0)<br>grid = GridSearchCV(rf,grid_params,cv = 10)	0.5749<br>max_features='auto'
Step 5	max_depth (5,6,7,8,9,10,11,12,13,14)<br>min_samples_leaf (1,2,3)<br>min_samples_split (2,3,4,5,6)<br>max_features ('auto', 'sqrt')	grid_params = {'max_depth':np.arange(5,15), 'min_samples_leaf':np.arange(1,4), 'min_samples_split':np.arange(2,7), 'max_features':['auto', 'sqrt']}<br>rf = ExtraTreesRegressor(n_estimators=34, random_state=0)<br>grid = GridSearchCV(rf,grid_params,cv = 10)	0.5786<br>max_depth=11<br>min_samples_leaf=1<br>min_samples_split=3<br>max_features='auto'

* The optimal feature set [including daily dosage of valproic acid, age, enzyme inducers, body weight, daily dosage of oxcarbazepine, and sex (male)] was determined before parameter adjustment.

Table S17 Algorithms, the corresponding packages, and tuning parameters in the Anaconda software used for each regression technique.

Algorithm	Package	Parameters tuned (default settings)
MLR	Sklearn 0.20.2 (from sklearn.linear_model import LinearRegression)	'copy_X': True, 'fit_intercept': True, 'n_jobs': None, 'normalize': False
RidgeR	Sklearn 0.20.2 (from sklearn.linear_model import Ridge)	'alpha': 1.0, 'copy_X': True, 'fit_intercept': True, 'max_iter': None, 'normalize': False, 'random_state': None, 'solver': 'auto', 'tol': 0.001
LassoR	Sklearn 0.20.2 (from sklearn.linear_model import Lasso)	'alpha': 1.0, 'copy_X': True, 'fit_intercept': True, 'max_iter': 1000, 'normalize': False, 'positive': False, 'precompute': False, 'random_state': None, 'selection': 'cyclic', 'tol': 0.0001, 'warm_start': False
LSVR	Sklearn 0.20.2 (from sklearn.svm import LinearSVR)	'C': 1.0, 'dual': True, 'epsilon': 0.0, 'fit_intercept': True, 'intercept_scaling': 1.0, 'loss': 'epsilon_insensitive', 'max_iter': 1000, 'random_state': None, 'tol': 0.0001, 'verbose': 0
ETR	Sklearn 0.20.2 (from sklearn.ensemble import ExtraTreesRegressor)	'bootstrap': False, 'criterion': 'mse', 'max_depth': None, 'max_features': 'auto', 'max_leaf_nodes': None, 'min_impurity_decrease': 0.0, 'min_impurity_split': None, 'min_samples_leaf': 1, 'min_samples_split': 2, 'min_weight_fraction_leaf': 0.0, 'n_estimators': 'warn', 'n_jobs': None, 'oob_score': False, 'random_state': None, 'verbose': 0, 'warm_start': False
KNR	Sklearn 0.20.2 (from sklearn.neighbors import KNeighborsRegressor)	'algorithm': 'auto', 'leaf_size': 30, 'metric': 'minkowski', 'metric_params': None, 'n_jobs': None, 'n_neighbors': 5, 'p': 2, 'weights': 'uniform'
BR	Sklearn 0.20.2 (from sklearn.ensemble import BaggingRegressor)	'base_estimator': None, 'bootstrap': True, 'bootstrap_features': False, 'max_features': 1.0, 'max_samples': 1.0, 'n_estimators': 10, 'n_jobs': None, 'oob_score': False, 'random_state': None, 'verbose': 0, 'warm_start': False
RFR	Sklearn 0.20.2 (from sklearn.ensemble import RandomForestRegressor)	'bootstrap': True, 'criterion': 'mse', 'max_depth': None, 'max_features': 'auto', 'max_leaf_nodes': None, 'min_impurity_decrease': 0.0, 'min_impurity_split': None, 'min_samples_leaf': 1, 'min_samples_split': 2, 'min_weight_fraction_leaf': 0.0, 'n_estimators': 'warn', 'n_jobs': None, 'oob_score': False, 'random_state': None, 'verbose': 0, 'warm_start': False
XGBR	Xgboost 0.80 (from xgboost import XGBRegressor)	'base_score': 0.5, 'booster': 'gbtree', 'colsample_bylevel': 1, 'colsample_bytree': 1, 'gamma': 0, 'importance_type': 'gain', 'learning_rate': 0.1, 'max_delta_step': 0, 'max_depth': 3, 'min_child_weight': 1, 'missing': None, 'n_estimators': 100, 'n_jobs': 1, 'nthread': None, 'objective': 'reg:linear', 'random_state': 0, 'reg_alpha': 0, 'reg_lambda': 1, 'scale_pos_weight': 1, 'seed': None, 'silent': True, 'subsample': 1
GBR	Sklearn 0.20.2 (from sklearn.ensemble import GradientBoostingRegressor)	'alpha': 0.9, 'criterion': 'friedman_mse', 'init': None, 'learning_rate': 0.1, 'loss': 'ls', 'max_depth': 3, 'max_features': None, 'max_leaf_nodes': None, 'min_impurity_decrease': 0.0, 'min_impurity_split': None, 'min_samples_leaf': 1, 'min_samples_split': 2, 'min_weight_fraction_leaf': 0.0, 'n_estimators': 100, 'n_iter_no_change': None, 'presort': 'auto', 'random_state': None, 'subsample': 1.0, 'tol': 0.0001, 'validation_fraction': 0.1, 'verbose': 0, 'warm_start': False
DTR	Sklearn 0.20.2 (from sklearn.tree import DecisionTreeRegressor)	'criterion': 'mse', 'max_depth': None, 'max_features': None, 'max_leaf_nodes': None, 'min_impurity_decrease': 0.0, 'min_impurity_split': None, 'min_samples_leaf': 1, 'min_samples_split': 2, 'min_weight_fraction_leaf': 0.0, 'presort': False, 'random_state': None, 'splitter': 'best'
SVR	Sklearn 0.20.2 (from sklearn.svm import SVR)	'C': 1.0, 'cache_size': 200, 'coef0': 0.0, 'degree': 3, 'epsilon': 0.1, 'gamma': 'auto_deprecated', 'kernel': 'rbf', 'max_iter': -1, 'shrinking': True, 'tol': 0.001, 'verbose': False
NuSVR	Sklearn 0.20.2 (from sklearn.svm import NuSVR)	'C': 1.0, 'cache_size': 200, 'coef0': 0.0, 'degree': 3, 'gamma': 'auto_deprecated', 'kernel': 'rbf', 'max_iter': -1, 'nu': 0.5, 'shrinking': True, 'tol': 0.001, 'verbose': False
MLPR	Sklearn 0.20.2	'activation': 'relu', 'alpha': 0.0001, 'batch_size': 'auto', 'beta_1': 0.9, 'beta_2': 0.999, 'early_stopping': False, 'epsilon': 1e-08, 'hidden_layer_sizes': (100,), 'learning_rate': 'constant', 'learning_rate_init': 0.001, 'max_iter': 200, 'momentum': 0.9,

	(from sklearn.neural_network import MLPRegressor)	'n_iter_no_change': 10, 'nesterovs_momentum': True, 'power_t': 0.5, 'random_state': None, 'shuffle': True, 'solver': 'lbfgs', 'tol': 0.0001, 'validation_fraction': 0.1, 'verbose': False, 'warm_start': False
ABR	Sklearn 0.20.2 (from sklearn.ensemble import AdaBoostRegressor)	'base_estimator': None, 'learning_rate': 1.0, 'loss': 'linear', 'n_estimators': 50, 'random_state': None

Abbreviations: MLR, multiple linear regression; RidgeR, ridge regression; LassoR, lasso regression; LSVR, linear-support vector regression; ETR, extra-trees' regression; KNR, k-nearest neighbor regression; BR, bagging regression; RFR, random forest regression; XGBR, XGBoost regression; GBR, gradient boosted regression; DTR, decision tree regression; SVR, support vector regression; NuSVR, nu-support vector regression; MLPR, multi-layer perceptron regression; ABR, AdaBoost regression.
